# Rapid metagenomic sequencing for diagnosis and antimicrobial sensitivity prediction of canine bacterial infections

**DOI:** 10.1099/mgen.0.001066

**Published:** 2023-07-20

**Authors:** Natalie Ring, Alison S. Low, Bryan Wee, Gavin K. Paterson, Tim Nuttall, David Gally, Richard Mellanby, J. Ross Fitzgerald

**Affiliations:** ^1^​ The Roslin Institute, University of Edinburgh, Edinburgh, UK; ^2^​ Royal (Dick) School of Veterinary Studies, University of Edinburgh, Edinburgh, UK

**Keywords:** nanopore sequencing, whole genome sequencing, rapid diagnostics, AMR, infection

## Abstract

Antimicrobial resistance is a major threat to human and animal health. There is an urgent need to ensure that antimicrobials are used appropriately to limit the emergence and impact of resistance. In the human and veterinary healthcare setting, traditional culture and antimicrobial sensitivity testing typically requires 48–72 h to identify appropriate antibiotics for treatment. In the meantime, broad-spectrum antimicrobials are often used, which may be ineffective or impact non-target commensal bacteria. Here, we present a rapid, culture-free, diagnostics pipeline, involving metagenomic nanopore sequencing directly from clinical urine and skin samples of dogs. We have planned this pipeline to be versatile and easily implementable in a clinical setting, with the potential for future adaptation to different sample types and animals. Using our approach, we can identify the bacterial pathogen present within 5 h, in some cases detecting species which are difficult to culture. For urine samples, we can predict antibiotic sensitivity with up to 95 % accuracy. Skin swabs usually have lower bacterial abundance and higher host DNA, confounding antibiotic sensitivity prediction; an additional host depletion step will likely be required during the processing of these, and other types of samples with high levels of host cell contamination. In summary, our pipeline represents an important step towards the design of individually tailored veterinary treatment plans on the same day as presentation, facilitating the effective use of antibiotics and promoting better antimicrobial stewardship.

## Data Summary

All sequencing data mentioned in this work is available from NCBI, BioProject PRJNA925092, Biosamples SAMN32880396 to SAMN32880438, run accessions SRR23195371 to SRR23195413.

This includes 35 samples sequenced during the development of the protocol (full details in Table S2, available in the online version of this article, ‘Protocol Development’) and eight samples sequenced during the real-time processing phase with the finalised protocol (full details in Table S7, ‘Realtime clinical samples’). Samples from which no DNA could be extracted were not sequenced during either phase, therefore no data is available for them from the NCBI.

Source code and full commands used for mentioned tools are available from Github: https://github.com/nataliering/Dogstails.

The authors confirm all supporting data, code and protocols have been provided within the article or through supplementary data files.

**Impact Statement**

Antimicrobial resistance (AMR) is a major threat to veterinary and human healthcare. It is a One Health problem, as humans and dogs are in close contact, require similar antibiotics, and share bacterial pathogens and AMR genes. Limited treatment options due to AMR would have a catastrophic effect. The risk of infection would render much of modern healthcare (including critical care, orthopaedic and complex surgeries, implants and oncology) impossible. In addition, routine infections could become life threatening. It is therefore critical to preserve the efficacy of these drugs for the future. Inappropriate antimicrobial use is the single biggest factor driving AMR. Antimicrobial stewardship involves reducing antimicrobial use, using first-line narrow-spectrum drugs, and avoiding overly long treatment. Delays in culture-based diagnosis lead clinicians to speculatively use broad-spectrum antibiotics and prolong courses of treatment beyond clinical cure. Sequencing-based rapid diagnostic approaches will have a major impact in reducing, refining and replacing antibiotic use. Such techniques will advance antimicrobial stewardship in veterinary and human healthcare.

## Introduction

Antimicrobial resistance (AMR) levels among human and veterinary bacterial pathogens are escalating globally, to the extent that the World Health Organization now classifies AMR as one of the biggest threats to global health, food security and development [1, 2]. The current gold-standard diagnostic methods used in human and veterinary practice are culture-based or involve remote service providers for PCR, requiring several days to yield results. Broad-spectrum or inappropriate antibiotics are often started while waiting for the results; this inappropriate antimicrobial use is a major driver for AMR [3]. Furthermore, animals with suspected highly contagious or potentially zoonotic infections like leptospirosis may require quarantining while waiting for a diagnosis, leading to escalated costs, wasted resources, and unnecessary stress for the quarantined animal [4].

Novel methods that enable sensitive, same-day rapid diagnosis and prediction of antibiotic resistance across different infection types are required. Targeted molecular tests, including PCR panels of selected AMR genes, are one option already in use [5, 6]. However, targeted tests often require prior knowledge (or suspicion) of the causative pathogen, whilst gene panels may risk missing AMR caused by unexpected or novel mechanisms. Likewise, strategies involving metabarcoding for the identification of bacteria in human clinical samples, such as 16S rRNA gene amplification and sequencing, have been thoroughly tested and reviewed elsewhere [7–13]. These studies have often used Oxford Nanopore Technologies’ (ONT) long read sequencing technology, which allows rapid sequencing and real-time data analysis, and have shown the ability to accurately identify bacteria, usually to the species level, despite the historically higher error rates produced by the technology. However, although full-length 16S sequencing with long reads may enable accurate species identification, the 16S gene gives no information about potential AMR phenotypes present in the present species.

An alternative method is therefore metagenomic whole genome sequencing (mWGS), involving the extraction of all genomic DNA (gDNA) present in a sample and subsequent identification of pathogens by unbiased DNA sequencing. Such methods would also use ONT sequencers, due to their rapid turnaround time. The ONT platforms most suited for this kind of rapid diagnosis are the MinION and GridION, which have already been tested with mWGS for human clinical sputum, endotracheal aspirate, blood, urine and other sample types [14–23]. The MinION’s small size (similar to a small smartphone for the Mk1B or a handheld games console for the Mk1C) and relatively low start-up costs could enable its usage in a wide variety of clinical settings. In contrast, the GridION is larger (requiring around as much bench space as two desktop computers plus their monitors) and more expensive but may be suited to a larger hospital environment where more samples are processed. Indeed, the MinION and GridION are already both in use in some NHS hospitals [24, 25].

With both the MinION and the GridION, sequence data is accessible in real-time as it is produced, potentially reducing the time required to identify pathogens and their AMR genotypes to as little as 10 min [26, 27]. Furthermore, the Flongle is an adaptor which fits into MinION and GridION sequencing devices, and enables the use of lower throughput, single-use Flongle flow cells, which may offer a more cost-effective solution which is suited to a clinical environment [14, 28]. The long reads produced by nanopore sequencing also readily facilitate whole genome assembly which potentially allows the linkage of AMR genes to specific bacterial strains [29]. Whilst many previous studies have focussed on human clinical applications, Nanopore sequencing (isolate, metagenomic and 16S) has also already been used in a veterinary context, for example to examine the spread of AMR genes within a veterinary hospital, and to investigate a wide variety of veterinary pathogens [30–37].

Our primary objective here was to develop a rapid, culture-free mWGS pipeline to identify pathogens and predict AMR in canine samples in a veterinary hospital setting. This included selecting and validating an extraction protocol, determining what additional clean-up steps might be required for clinical samples, determining which sequencing library preparation method produced the most reliable results, determining the most cost-effective way to use sequencing flow cells in a clinical setting where sample numbers might be inconsistent, and testing how the finalised protocol performed when compared to the current gold standard culture-based clinical microbiology techniques, taking into account the accuracy of the sequencing-based predictions as well as the time taken to produce them.

To initially develop our pipeline, we focus here on two common canine infections: urinary tract infections (UTIs) and skin infections (pyoderma). Antibiotic therapy is often the first line of treatment in these infections, but AMR, including multi-drug resistance, is frequently observed, and increasing in prevalence [38–42]. Furthermore, recurrent infections are common, leading to frequent return visits to the clinic and further courses of antibiotics [43, 44]. A rapid, sensitive method for diagnosing the bacterial pathogens in urine and skin swab samples, and predicting their antimicrobial sensitivity, could therefore prevent the use of inappropriate antibiotics, and limit the amount of clinical care required.

## Methods

Source code and full commands used for mentioned data analysis tools are available from Github: https://github.com/nataliering/Dogstails

### Optimisation of DNA extraction

Our first objective was to develop and test a DNA extraction protocol suitable for both urine and skin swab samples, and ideally adaptable to additional sample types in the future, ending with DNA of suitable quality for sequencing library preparation. We examined the records from the University of Edinburgh’s Hospital for Small Animals covering a 2 year period (2018 and 2019) to determine which species were most commonly seen in urinary tract and skin infections, and aimed to develop a protocol which would be capable of producing sequencing-quality DNA from them all. This objective included several steps: selection of a DNA extraction kit, selection and optimisation of a metagenomic lysis step to incorporate into the selected kit, validating that the selected lysis method and kit could extract DNA from the most commonly seen species in the HfSA urine and skin records, and determining whether additional post-extraction steps would be required.

#### Selection of DNA extraction kit

Three different kits recommended in the literature were tested: DNeasy Blood + Tissue (Qiagen, Hilden, Germany), DNeasy Powersoil (Qiagen) and MagAttract HMW DNA (Qiagen). To test each kit, overnight *Escherichia coli* CAN-50 growth in Luria-Bertani (LB, ThermoFisher, Massachusetts, USA) broth equivalent to 10^9^ CFUs was spun down at 16 000 ***g***, and the cell pellet was resuspended in 1 ml healthy dog urine. The urine-cell suspension was then processed using each kit, according to the relevant manufacturer’s instructions for each, finishing with an elution into 50 µl nuclease-free water. For each extraction, two negative controls were used: 3 ml of uncultured broth and 160 µl 50 mM Tris, 10 mM EDTA, pH 8.0 (buffer P1 as per the MagAttract HMW DNA protocol). The resulting extractions were used to compare the kits in terms of (i) gDNA yield in 50 µl nuclease-free water (quantified by Qubit dsDNA HS kit), (ii) lysis method for Gram-positive species (enzymatic vs bead-beating), (iii) speed, and (iv) cost.

#### Optimisation of metagenomic bacterial lysis

Records for canine urine and skin swab samples processed at the Royal (Dick) School of Veterinary Studies Hospital for Small Animals (HfSA) in Edinburgh between 2018 and 2019 were analysed to determine which species were most commonly detected, so the broad efficacy of our extraction protocol for the most relevant pathogens could be tested (Table S1). Metapolyzyme (Sigma-Aldrich, Missouri, USA), an enzymatic lysis cocktail containing six lysis enzymes (achromopeptidase, chitinase, lyticase, lysostaphin, lysozyme and mutanolysin), was trialled for our extraction protocol. An isolate of the Gram-positive species *Staphylococcus pseudintermedius *, ED99 [45], was used to trial four enzymatic lysis options: lysostaphin, lysozyme, and two different concentrations of metapolyzyme. Cells were grown overnight on tryptic soy agar (TSA, Oxoid ThermoFisher, Massachusetts, USA) plates at 37 °C, then a single colony was transferred into tryptic soy broth (TSB, Oxoid) media and cultured overnight at 37 °C with shaking. 2.5 ml of overnight culture was pelleted by centrifuging for 3 min at 16 000 ***g***, then resuspended in 3 ml healthy dog urine. Two negative control samples were used: 3 ml of the same healthy dog urine, and 160 µl 50 mM Tris, 10 mM EDTA, pH 8.0 (buffer P1 as per the MagAttract HMW DNA protocol).

For each of the four lysis options tested, the resulting urine-and-cell suspension was centrifuged for 3 min at 16 000 ***g***, then resuspended in 160 µl 50 mM Tris, 10 mM EDTA, pH 8.0 (buffer P1 for the MagAttract HMW DNA kit). Then 20 µl lysozyme (100 mg ml^−1^), lysostaphin (10 mg ml^−1^), metapolyzyme (6.6 mg ml^−1^) or metapolyzyme (3.3 mg ml^−1^) was added, and the solution mixed by flicking. The solution was then incubated on a thermomixer for 1 h at 37 °C with 900 r.p.m. shaking. After 1 h, 20 µl proteinase K was added, and the solution was incubated for a further 30 min at 56 °C with 900 r.p.m. shaking. The rest of the MagAttract HMW DNA Gram-positive protocol was then followed as per the manufacturer’s instructions (starting from step 8 on page 26 in the MagAttract HMW DNA Handbook 03/2020), eluting into 50 µl nuclease-free water as the final step. DNA concentrations were quantified using the Qubit dsDNA HS kit according to the manufacturer’s instructions.

#### Testing our extraction protocol on different species

The optimised MagAttract + Metapolyzyme extraction protocol was tested on the most commonly isolated species identified in the HfSA’s records: *E. coli *, *S. pseudintermedius *, *Streptococcus canis *, *Enterococcus faecalis *, *Pseudomonas aeruginosa *, *Proteus mirabilis *, *Pasteurella canis *, *Klebsiella pneumoniae *, *Kocuria kristinae/Kocuria rosea*, and *Clostridium perfringens *. For aerobic species, cells were grown on TSA plates at 37 °C for 24 h or 72 h (*Kocuria/Streptococcus*), then in TSB medium at 37 °C with shaking for 24 h or 72 h (* Kocuria */* Streptococcus *). For two anaerobic or facultative anaerobic species (* C. perfringens* and *P. canis *), cells were grown on TSA plates at 37 °C in a sealed box with Anaerogen sachets (Oxoid ThermoFisher) for 24 h, then in TSB medium in growth flasks in a sealed box with Anaerogen sachets at 37 °C with shaking for 24 h. For all species, 3 ml of broth culture was pelleted by centrifuging at 16 000 ***g*** for 3 min, the pellet was resuspended in 3 ml healthy dog urine, then pelleted again in the same way. Again, 3 ml the healthy dog urine and 160 µl buffer P1 were also used as a negative control. Cell pellets was then processed as per the optimised protocol, starting with resuspension in 160 µl buffer P1 and 60 min lysis with 20 µl metapolyzyme. DNA was eluted into 50 µl nuclease-free water, and quantified using Qubit’s dsDNA HS kit.

#### Determining whether additional post-extraction DNA clean-up was required

The purity of the extracted DNA was assessed using a Nanodrop spectrophotometer (ThermoFisher) to measure the 260/280 nm and 260/230 nm absorbance ratios of 21 clinical samples (a selection of the total samples processed, indicated in Table S2. The samples were then cleaned up using the ProNex Size-Selective Purification System (Promega, Wisconsin, USA) according to manufacturer’s instructions, starting with 50 µl DNA and 200 µl ProNex beads, and eluting into 20 µl nuclease-free water at the end of the protocol. The purity of the cleaned-up DNA was then measured again by Nanodrop. The differences in purity of the pre- and post-clean-up samples were assessed for significance using the Wilcoxon test for paired samples. After these 21 samples, the ProNex clean-up was incorporated as standard, and for reasons of speed, Nanodrop readings were no longer taken.

#### Final optimised protocols for metagenomic DNA extraction and clean-up

The final optimised protocols, including post-extraction clean-up with ProNex beads, are available from protocols.io: https://www.protocols.io/view/magattract-metapolyzyme-metagenomic-gdna-extractio-n2bvj8o5bgk5/v2 (urine) https://www.protocols.io/view/magattract-metapolyzyme-metagenomic-gdna-extractio-q26g7yr19gwz/v2 (skin swabs).

#### Optimisation of the sequencing process

After developing our metagenomic DNA extraction protocol for clinical samples, our next objective was to optimise the sequencing process. Throughout the development of this process, we needed to assess the taxonomic composition of our sequenced DNA, hence we first selected two commonly used bioinformatics taxonomic classification tools to use throughout this phase. The remaining steps in the sequencing optimisation phase included: selection of a library preparation kit, testing the lower limits of the selected kit, comparison and selection of flow cell type (MinION vs Flongle), and developing the optimal usage strategy for the selected flow cell type.

#### Species identification strategies

For each experiment detailed here, species were identified from sequencing reads using either EPI2ME workflows ‘Fastq WIMP (What’s in my pot?)’ and ‘Fastq Antimicrobial Resistance’ (v3.0.1–7052513, https://epi2me.nanoporetech.com/, ONT account required), Kraken2 (v2.1.1, [46]) with one of two custom databases, ‘pathogens_plus’ or ‘bacteria_plus’, or a combination thereof. EPI2ME is ONT’s own software, designed to be used with uncorrected nanopore long reads. The WIMP workflow classifies reads using the Centrifuge standard database, whilst the Antimicrobial Resistance workflow uses minimap2 to align input reads to the AMR CARD database to identify AMR genes.

The custom Kraken2 bacteria_plus database was constructed from all bacterial representative genomes present in the NCBI RefSeq database in November 2022 (16 113 species) plus eight mammalian genomes (*Canis lupus familiaris*, *Homo sapiens*, *Felis catus*, *Equus caballus*, *Oryctolagus cuniculus*, *Sus domesticus*, *Bos taurus* and *Ursus arctos*). The ‘pathogens_plus’ database contains 668 genomes of various bacterial, viral, protozoan and fungal pathogens, including the 100 common human and animal pathogens identified in a 2014 study [47], plus selected other pathogens known to be important in veterinary samples, such as *Leptospira* and *Mycobacterium* spp. (a full list of species included can be seen in Table S3). The pathogens_plus database also contains the same eight mammalian genomes included in the bacteria_plus database. The two Kraken2 databases were tested using two ZymoBIOMICS Microbial Community Standards datasets (log and even distributions of species) sequenced on R9.4.1 flow cells by Nicholls *et al*. in 2019 [48], as well as single isolate datasets for the same ten most commonly isolated species tested in the ‘Testing our extraction on different species’ section above. Full details of these tests and their results are given in Supplementary Results SR1.

The tool and database used is noted for each step of the protocol development below, with further details as necessary. Negative control samples (unless otherwise stated, nuclease-free water which was subjected to the entire same protocol as the test samples) were sequenced to characterise any background contamination and any barcode-crosstalk (when sequencing multiple samples simultaneously). When testing clinical samples, a threshold of ≥1 % of classified bacterial reads was used to determine true positives and minimise false positives arising from any remaining background contaminants and low quality database alignments.

#### Selection of a library preparation kit

Two rapid library preparation kits were tested: rapid PCR barcoding (SQK-RPB004, Oxford Nanopore Technologies (ONT), Oxford, UK) and rapid barcoding (SQK-RBK004, ONT). The SQK-RPB004 PCR reaction was carried out on 22 clinical samples (Table S2), according to the manufacturer’s instructions, and the concentration of each was measured by Qubit’s dsDNA HS kit before and after the reaction.

All 22 samples prepared with the rapid PCR barcoding kit were subsequently sequenced on MinION R9.4.1 flow cells (ONT) according to the SQK-RPB004 protocol, although ProNex beads were substituted into steps which required AMPure XP beads. Four of these samples, two urines (DTU09 and DTU16) and two skin swabs (SkSw08A and SkSw14), were re-sequenced with the rapid barcoding kit, to compare the results of sequencing with and without the PCR amplification. The DNA was prepared according to the manufacturer’s instructions for SQK-RBK004, except for the substitution of ProNex in place of AMPure XP again. For both SQK-RPB004 and SQK-RBK004, the sequencing was performed using a MinION Mk1C device running either MinKNOW v19.12.12 or v21.05.12 (see Table S2) and basecalling was performed after sequencing using the University of Edinburgh’s high-performance computing cluster (Eddie) to run GPU Guppy (v5.0.7) in super accuracy (SUP) mode. The results of the two library preparation methods were compared in terms of (i) run yield, and (ii) bacterial reads vs eukaryotic host contamination (according to ONT’s online EPI2ME classification ‘WIMP’ tool).

#### Determining the lower detection limits for the rapid barcoding (SQK-RBK004) kit

To estimate the lowest DNA concentration at which species could still be identified, a sequencing run was conducted using serially diluted gDNA. The gDNA was a 50 : 50 mix of *S. pseudintermedius* and dog DNA. Both were extracted using the MagAttract HMW kit (* S. pseudintermedius* from overnight TSB culture, dog from a surplus diagnostic blood sample from the HfSA’s clinical biobank, as per the MagAttract protocol for blood), cleaned-up with ProNex beads as described above, quantified using Qubit’s dsDNA HS kit and adjusted to 75 ng µl^−1^ prior to mixing. The mixed sample was further diluted to a starting concentration of 10 ng µl^−1^ (confirmed by Qubit dsDNA HS kit), then the 10 ng µl^−1^ sample was serially 1 : 1 diluted in nuclease-free water, from around 10 ng µl^−1^ down to below 0.1 ng µl^−1^. Each dilution was prepared for sequencing with SQK-RBK004 as described above, with a different barcode used for each dilution and barcode01 used as a negative control (nuclease-free water). The dilution series plus negative control was then sequenced on a fresh MinION R9.4.1 flow cell for 24 h. We know from prior experience that 10 ng µl^−1^ sequences adequately using SQK-RBK004, hence this starting concentration acted as our positive control for this test. The sequencing data was analysed using Kraken2 with the bacteria_plus database. The results for the negative control sample were used to determine levels of background contamination and barcode crosstalk. The number of reads for each expected species (* S. pseudintermedius* and *C. lupus familiaris*) seen in the negative control (representing barcode crosstalk) were subtracted from the other samples, and the results for each dilution were compared in terms of (i) run yield, and (ii) reads from the two expected species.

#### Comparing MinION flow cells with Flongle flow cells

Two relatively highly concentrated gDNA samples were used to compare higher throughput MinION flow cells with single-use, low-throughput Flongle flow cells: a clinical skin swab sample (SkSw21, 55 ng µl^−1^) and a 1 : 1 mix of *E. coli* and *P. aeruginosa* isolate DNA, both extracted previously during the DNA extraction optimisation trials (80 ng µl^−1^). Each sample was prepared for sequencing on an R9.4.1 Flongle flow cell (FLO-FLG001) according to the Flongle-specific manufacturer’s instructions for SQK-RBK004 (using ProNex beads instead of AMPure XP). Each sample was then sequenced separately for a full 24 h with the Flongle adapter on a MinION Mk1C running MINKOW (v21.02.2) and real-time basecalling with Guppy (v4.3.4) in ‘fast’ mode in order to monitor sequence acquisition, and the total yield of the 24 h run was noted. More DNA from the same samples was then prepared for sequencing on an R9.4.1 MinION flow cell with SQK-RBK004 as above. Sequencing was started for the first (*E. coli + P. aeruginosa*) sample on a Mk1C MinION running MINKOW (v21.02.2) and real-time basecalling with Guppy (v4.3.4) in ‘fast’ mode in order to monitor sequence acquisition. The sequencing was stopped when the run yield matched that of the 24 h Flongle run, and the time taken to reach that yield was recorded. The MinION flow cell was then washed using the flow cell wash kit (EXP-WSH004, ONT) according to the manufacturer’s instructions, and the second sample (SkSw21) was loaded and sequenced using the same conditions. Again, the sequencing was monitored closely and stopped once the run yield matched that of the 24 h Flongle run, and the time taken to reach that yield was compared between the MinION and the Flongle flow cells.

#### Determining the optimal MinION flow cell usage strategy

As the theoretical yield of each MinION flow cell is much higher than required for a single clinical sample, and to improve the cost-effectiveness of using MinION flow cells, two flow cells and a variety of DNA samples were used to test how many times a MinION flow cell can be washed and re-used. For the first flow cell, the DNA sample was a relatively high concentration previously extracted *E. coli* isolate (110 ng µl^−1^) which had both been stored at 4 °C since extraction. An initial sample of 7.5 µl of DNA was prepared for sequencing with SQK-RBK004 as previously described, using barcode01. Sequencing commenced on a fresh flow cell (on a Mk1C MinION running MINKOW [v21.02.2] and real-time basecalling with Guppy [v4.3.4] in ‘fast’ mode in order to monitor sequence acquisition) and was stopped when a target of 200 Mbp was reached (we assumed at this stage that most real clinical samples would need a maximum of 200 Mbp for species identification and AMR prediction). The time taken to reach 200 Mbp was recorded, and the flow cell was washed using EXP-WSH004 as previously described.

A second 7.5 µl was processed in the same way, with barcode02, followed by another wash with EXP-WSH004. This protocol continued until the time taken to reach 200 Mbp was longer than 2 h, using the next subsequent barcode (barcode03, barcode04, etc.). For each sequencing step, the starting number of available sequencing pores was recorded at the beginning of the run. After each run, the sequenced reads were quality controlled by EPI2ME and NanoStat (v1.6.0, [49]) and re-basecalled using the University of Edinburgh’s high-performance computing cluster (Eddie) to run GPU Guppy (v5.0.7) in super accuracy (SUP) mode in order to accurately determine the percentage of DNA assigned to the wrong barcode.

For the second flow cell, the same protocol was followed, but freshly extracted DNA from clinical samples, ranging from 0.76 to >120 ng µl^−1^, was sequenced. Each sample was sequenced for up to 3 h, and some samples were sequenced simultaneously (with different barcodes). Full details of the samples sequenced are given in Table S4.

### Testing the optimised extraction and sequencing process on real clinical samples

After finalising our extraction and sequencing protocol, the objective of our final stage was to test it on real clinical samples, to determine how quickly we could produce results and how accurate our predictions were, compared to those produced by the current gold-standard culture-based techniques.

#### Sequencing clinical urine samples in real-time using the finalised protocol

To test the full optimised protocol in a realistic manner, a further nine ‘post-development’ urine samples were processed in real-time in batches of one to four using the finalised protocol. The post-development samples were collected from the HfSA and processed immediately using the finalised extraction protocol (https://www.protocols.io/view/magattract-metapolyzyme-metagenomic-gdna-extractio-chnrt5d6). The extracted DNA was prepared for sequencing using the rapid barcoding kit (SQK-RBK004) as described above, using barcode 01 for a negative control (nuclease-free water, which was processed using the same extraction protocol as the real samples to control for contaminating DNA from the extraction and barcode cross-contamination during sequencing) and the remaining sequential barcodes for the real samples. Samples were sequenced for up to 3 h, or until 100 Mbp had been sequenced, using an R9.4.1 MinION flow cell on a GridION device (MinKNOW version 22.10.5), with real-time Guppy super accurate basecalling (v6.3.8). Although we tested flow cell longevity by sequencing 200 Mb, we wanted to minimise sequencing time further here, hence testing the sequencing of only 100 Mb to determine if sufficient data was produced. In between sequencing runs, the flow cell was stopped, washed using the flow cell wash kit (EXP-WSH004) according to manufacturer’s instructions and stored with storage buffer at 4 °C until the next use.

#### Analysing the data produced by all of our samples, compared to culture-based results

During sequencing, the data produced underwent preliminary analysis using EPI2ME’s Fastq Antimicrobial Resistance workflow. After sequencing, the data were further analysed, including more accurate species identification with Kraken2 (with both the pathogens_plus and bacteria_plus databases) with the --confidence 0.05 flag. The results of running Kraken2 with both databases were manually compared, and a final call on predicted species was made (see Supplementary Results S1 for full details on this decision process).

After species prediction, reads were trimmed with Porechop (v0.4.2, [50]), genomes were assembled with with Flye (v2.9-b1774, [51]) and annotated using Prokka (v1.14.5, [52]). Further AMR prediction was carried out using Abricate (v1.0.1, [53]) with the NCBI database downloaded on 18 January 2022 and AMRFinderPlus (v3.10.45, [54]) with the database version 2022-10-11.2. The Comprehensive Antibiotic Resistance Database (CARD, https://card.mcmaster.ca/home) was consulted to manually relate any identified genes and mutations with their likely resistance phenotypes. The results from all data analyses were collated to predict which species had been present in the original sample, and the AMR phenotypes expected from them. In addition, the length of time taken for each sample was recorded. These were then compared with results from the HfSA’s Veterinary Pathology Unit (VPU), who processed the same nine samples at the same time, using their usual gold-standard culture-based techniques, including species identification and antibiotic susceptibility testing (AST) using a using a VITEK 2 (bioMérieux) instrument. The accuracy of our AMR predictions was compared to both ‘Resistant and Intermediate’ AST results, and ‘Resistant’ alone.

During the development of this protocol, 44 clinical samples were processed (in addition to the final nine ‘post-development’ samples; 23 of these had paired results from the VPU. To more extensively test our current bioinformatics pipeline, these 23 samples with paired VPU results were also processed using the same data analysis strategy as the post-development samples (Kraken2, Flye, Prokka, ABRicate and AMRFinderPlus). For some of these samples volumes of data much larger than 100 Mb had been sequenced. These large datasets were randomly down-sampled to 100 Mbp (for reasons detailed above) using Rasusa (v0.6.1, [55]) before data analysis. The results for these samples were then compared retrospectively to VPU results (see Table S6 for full sample details and VPU results).

## Results and discussion

### Optimisation of the extraction process

**Fig. 1.**
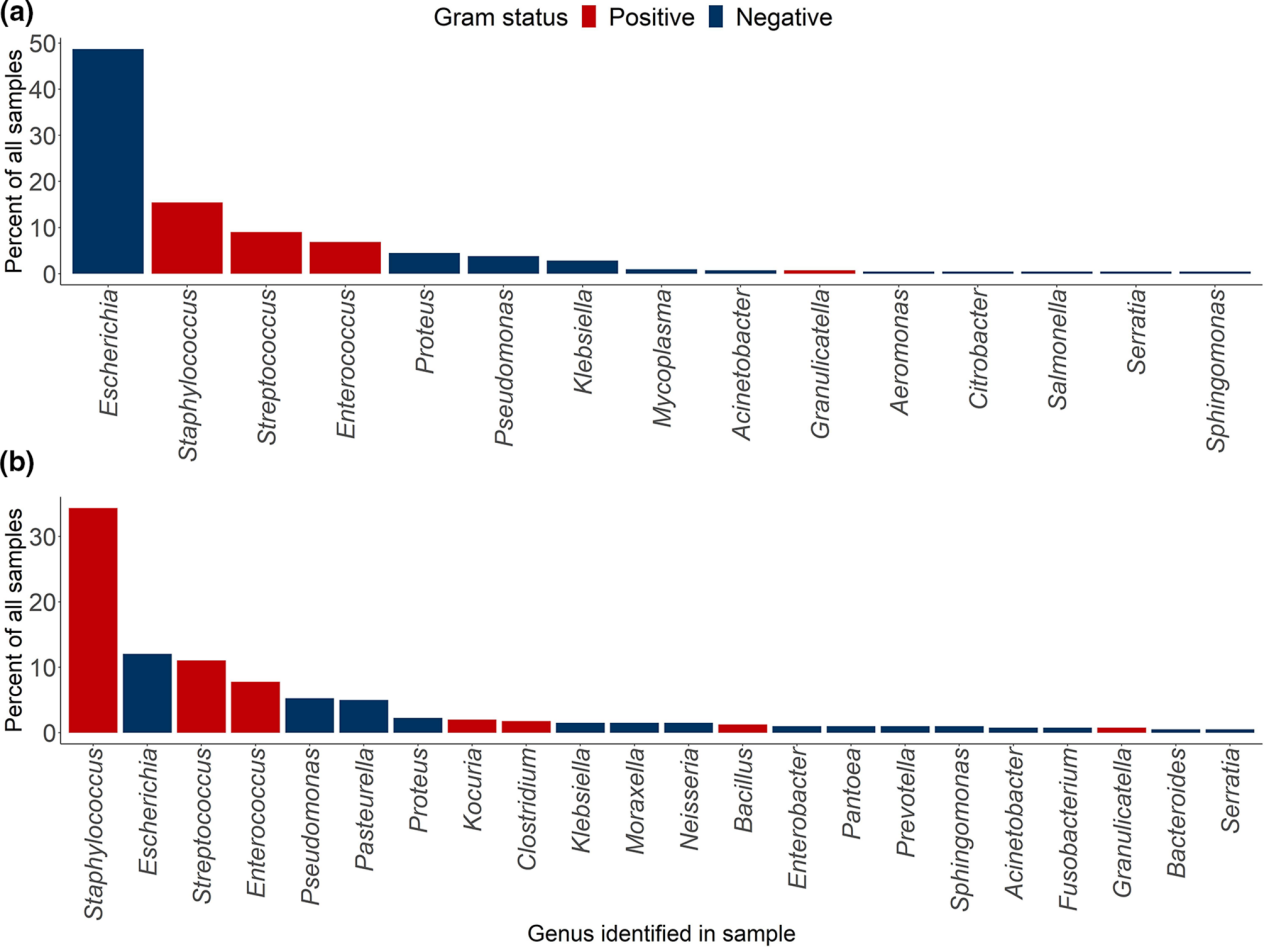
Pathogens identified in HfSA (A) urine and (B) skin swab samples, 2018 & 2019. Species observed in only one sample (17 species for urine samples, 22 for skin swab samples) are not shown here, but can be seen in Table S1.

As mentioned in the Methods section, the main objective for this stage of our project was to develop and test a DNA extraction protocol suitable for both urine and skin swab samples, which would be capable of producing sequencing-quality DNA from the vast majority of pathogens seen in urinary tract and skin infections. The development of this extraction protocol include: selection of a DNA extraction kit, optimisation of metagenomic lysis, validation that the selected lysis method and kit could extract DNA from the most commonly seen pathogens in the HfSA urine and skin records (Fig. 1), and determination of required additional post-extraction clean-up steps.

#### The MagAttract HMW kit can extract micrograms of DNA in around 30 minutes

A wide variety of DNA extraction methods are available, and many of them have been used in previous studies aiming to develop rapid diagnostic protocols or to extract metagenomic DNA from urine samples [16, 18, 20, 26, 29, 56–60]. We wanted to use a kit-based approach to increase consistency between samples, especially if the extraction was being performed by different individuals. We selected three commonly used kits to test (Table 1), based on their potential flexibility for application to other sample types in the future. A metagenomic approach using enzymatic lysis rather than mechanical cell disruption should result in high molecular weight (HMW) fragments, facilitating better genome assembly and more efficient species and AMR gene identification. Nonetheless, we tested one kit using mechanical disruption, to compare the efficiency of mechanical versus enzymatic lysis.

**Table 1.** gDNA extraction kits tested, and their pros and cons

Kit	Pros	Cons	Yield from test *E. coli* extraction (µg)	Cost per sample (kit only)
Qiagen DNeasy Blood + Tissue	Quick extraction (<40 min after lysis) Easy, column-based No additional reagents or equipment required No bead-beating	Different Gram +ve protocol (enzymatic lysis step required)	3.16	£3.92
Qiagen DNeasy PowerSoil	Very quick extraction (30 min or less) Same protocol for Gram +ve or –ve, optimised for metagenomics extractions	Extremely low yield Bead-beating, highly sheared DNA Not optimised for liquid samples	0.6	£6.06
Qiagen MagAttract HMW DNA	Optimised for extraction of HMW DNA No bead-beating Magnetic beads and six wash steps, so theoretically very clean DNA even from dirty starting samples Quick extraction (30 min after lysis)	Different Gram +ve protocol (enzymatic lysis step required) Magnetic rack required	3.08	£4.75

We tested three kits in the same way: attempting to extract cultured bacteria spiked into healthy dog urine, with the same healthy dog urine without spiked-in bacteria as a negative control. Although we tested the extractions with *E .coli*, we used the standard Gram-positive protocol (with lysozyme) for each kit, as our eventual protocol would need to efficiently extract DNA from both Gram-positive and Gram-negative species. Of the three kits we trialled, the one with mechanical lysis (the DNeasy Powersoil) was the most expensive per-sample, and produced lowest yield of gDNA when tested (Table 1). The two kits that included optional enzymatic Gram-positive lysis steps were the DNeasy blood + tissue kit and the MagAttract HMW kit, each producing similar yields of DNA in a similar time-frame when tested with *E. coli* (3.16 µg in 40 min vs 3.08 µg in 30 min, respectively). None of the kits we trialled produced levels of ‘kitome’ DNA that were detectable in our negative controls by the Qubit dsDNA HS kit, hence we assumed them all to contain similar (low) levels of background contamination [61]. Although the spin column-based DNeasy kit had a lower cost-per-sample, we ultimately selected the MagAttract for our protocol due to its optimisation towards extracting HMW DNA, slightly shorter extraction time, and its extensive washing steps that should limit the presence of inhibitors that affect library preparation.

#### Enzymatic lysis with 3.3 mg ml^−1^ metapolyzyme can produce micrograms of extracted DNA

We examined records from samples seen at the HfSA in 2018 and 2019 to assess the variety of bacterial pathogens associated with clinical urine or skin swab samples from dogs (Figs 1 and S1). Overall, around 90 % of culture-positive cases were associated with ten different genera, with a total of 53 different genera in total, including 48 % Gram-positive and 52 % Gram-negative (Table S1). We therefore required a lysis method that would allow the extraction of DNA from a wide variety of different species. As discussed above, we preferred enzymatic lysis over physical methods, due to the high levels of DNA shearing caused by physical methods. However, a single lysis enzyme such as lysozyme would not be capable of lysing the variety of Gram-positive bacteria seen in urine and skin infections.

Metapolyzyme is a cocktail of six different enzymes for the lysis of bacterial and/or fungal cell walls. Of these six, lysostaphin, mutanolysin and lysozyme should lyse all of the Gram-positive species most commonly seen in the 2018–2019 HfSA data. We assessed the efficiency of two concentrations of metapolyzyme compared to lysozyme and lysostaphin by lysing a cultured isolate of *Staphylococcus pseudintermedius* (the most common Gram-positive species in skin swabs) for 60 min, followed by DNA extraction with the MagAttract HMW kit selected above. We found that 3.3 mg ml^−1^ of metapolyzyme resulted in better yield of extracted DNA than either lysozyme or lysostaphin used at their recommended concentrations (Table 2). Although the higher concentration of metapolyzyme (6.6 mg ml^−1^) resulted in a slightly higher mass of extracted DNA than the lower, the gain was minimal (5.5 µg vs 5.2 µg), and thus not thought to be worth the added cost of using twice the enzyme per reaction. We consequently incorporated a 60 min lysis step with 3.3 mg ml^−1^ metapolyzyme before DNA extraction with the MagAttract kit.

**Table 2.** Different lysis enzymes tested with *S. pseudintermedius* ED99 DNA extraction

Lysis enzyme	DNA yield (µg)
Lysozyme (100 mg ml^−1^)	0.46
Lysostaphin (10 mg ml^−1^)	2.75
Metapolyzyme (6.6 mg ml^−1^)	5.5
Metapolyzyme (3.3 mg ml^−1^)	5.2

#### Our optimised protocol can efficiently extract DNA from the bacterial pathogens most commonly identified in canine urinary tract and skin infections

To validate that it would work for a variety of species, the optimised MagAttract + Metapolyzyme extraction protocol was next tested on representatives of the top ten genera identified in urine and/or skin swab samples (Fig. 1). Each species was grown in appropriate media and spiked into 3 ml healthy dog urine to simulate a clinical sample. As shown in Table 3, our protocol was successful in extracting micrograms of DNA from all ten species tested, including anaerobes, Gram-positives, and slow-growing bacteria (e.g. *Kocuria *). Although it is technically feasible that the healthy dog urine used may contain host cells as well as the spiked-in bacterial cells, our negative controls (extraction from the healthy dog urine without spiked-in bacteria) did not contain levels of DNA detectable by the Qubit dsDNA HS kit. We therefore concluded that any host DNA in our samples was negligible, and the vast majority of the micrograms of DNA extracted from our test samples was bacterial.

**Table 3.** gDNA concentrations extracted from ten most commonly encountered species, using our optimised lysis and extraction protocol

Species	Mean DNA yield (µg) *(standard deviation)*
* Escherichia coli *	>6.00 (*±0*)
*** Staphylococcus pseudintermedius ***	5.35 (*±0.15*)
* Proteus mirabilis *	5.85 (*±0.15*)
* Pseudomonas aeruginosa *	2.4 (*±0.72*)
*** Enterococcus faecalis ***	2.74 (*±0.87*)
*** Streptococcus canis ***	2.68 (*±0.18*)
***Kocuria rosea/kristinae***	2.09 (*±0.21*)
* Pasteurella canis* *	>6.00 (*±0*)
* Clostridium perfringens* *	1.86 (*±0.31*)
* Klebsiella pneumoniae *	>6.00 (*±0*)

**Bold** indicates Gram-positive species.

*Cultured in anaerobic conditions.

According to the HfSA records, the ten genera tested here represented around 90 % of urine and skin swab samples; the remaining 10 % of samples included 43 additional genera, each of which was seen only once or twice over a 2 year period at the HfSA. Therefore, testing every genera seen in the records was unfeasible at this point. However, overall, these tests served as proof that our protocol is effective in extracting DNA from the vast majority, if not all, of the species encountered in clinical canine urine or skin swab samples.

#### A post-extraction clean-up improves DNA purity for clinical samples

As clinical samples may contain contaminants or inhibitors that impact library preparation or sequencing, we tested if a further clean-up step would enhance our protocol and help to prevent library preparation failure. Nanodrop 260/280 nm and 260/230 nm absorbance ratios were used to assess the purity of 21 clinical samples (14 urine, 7 skin swabs, Fig. S1) before and after a clean-up protocol using the ProNex Size-Selective Purification System. This kit was selected because it should maintain the high molecular weight of the DNA extracted by the MagAttract protocol, and for its affordability relative to the AMPure XP system.

Prior to the clean-up, neither the mean 260/280 nm ratio nor the mean 260/230 nm ratio across the 21 samples was ideal (mean=2.00 and mean=0.78, compared to the expected ratios for pure DNA of ~1.8 and 2.0–2.2, respectively [62]). This suggested that significant levels of contaminants remained after the DNA extraction. After the clean-up, the mean 260/280 nm ratio across the 21 samples was close to ideal at 1.86, although the difference between pre- and post-clean-up ratios was not significant (*P*=0.8, *n*=21). In contrast, the 260/230 nm ratios were significantly improved post-clean-up (*P*=0.007, *n*=21); the mean value of 1.36 was still not optimal, but it did enable efficient library preparation. Our findings support the inclusion of the additional clean-up step between DNA extraction and library preparation, despite adding extra time to the final length of the rapid diagnostics protocol. An additional benefit of the clean-up protocol is that by eluting into a final volume lower than the starting volume (20 µl vs 50 µl) the DNA is further concentrated.

#### Final DNA extraction protocol

Our final protocol for metagenomic DNA extraction from urine and skin swab samples is therefore a 60 min lysis with 3.3 mg ml^−1^ of metapolyzyme, 30 min DNA extraction with the MagAttract HMW kit, and 30 min DNA clean-up with ProNex beads, eluting into a concentrated 20 µl nuclease-free water.

### Optimisation of the sequencing process

After the development of our DNA extraction protocol, which outputs high quality DNA ready for sequencing, we next optimised the steps necessary to produce DNA sequencing reads. This included the selection of a sequencing library preparation kit, testing the limits of that library preparation kit, selecting a flow cell type, and determining the most cost-effective usage strategy for the selected flow cells.

#### Rapid barcoding enables sequencing and identification of species from extracted concentrations of DNA as low as 0.08 ng µl^−1^

Our experience extracting DNA from the first few clinical samples (Table S2) indicated that DNA concentrations may be relatively low in some samples (the mean concentration of the first five skin swab samples was just 4.86 ng µl^−1^, whilst the mean from the first five urine samples was 21.98 ng µl^−1^). Some previous protocols developed for rapid bacterial identification by whole genome nanopore sequencing have used ONT’s rapid PCR barcoding library preparation kit, SQK-RPB004 [16, 18, 63, 64]. The major benefit of this kit is the amplification of DNA during the PCR step, which may be important for low abundance samples, though with the disadvantage of the additional time required. We therefore tested this kit first, using it to attempt library preparation of DNA extracted from 27 samples (15 urine, 12 skin swabs), with pre-PCR DNA masses ranging from 1.95 to 8.7 ng. However, we found that the PCR step resulted in unpredictable yields of DNA (independent of starting mass), and a tendency to amplify host DNA more than bacterial (Figs S3 and S4). The failure of the PCR reaction for some samples was likely due to PCR inhibitors remaining even after a DNA clean-up step; whether or not inhibitors still remain is unpredictable as it seems to vary between samples. A second DNA clean-up step might result in more consistent PCR success, but it would add at least 30 min to our protocol, and PCR might still fail.

We therefore trialled ONT’s rapid barcoding kit without the PCR step (SQK-RBK004) instead. We used a serial dilution series of combined *S. pseudintermedius* and *C. lupus familiaris* DNA to determine the lowest concentration which could be sequenced and still produce enough usable data to identify selected bacterial species from our samples. A 50 : 50 ratio of bacterial to canine DNA was chosen, as this represents the average level of host contamination seen in our urine samples throughout development, and therefore was a realistic approximation of a low concentration clinical sample.

**Fig. 2.**
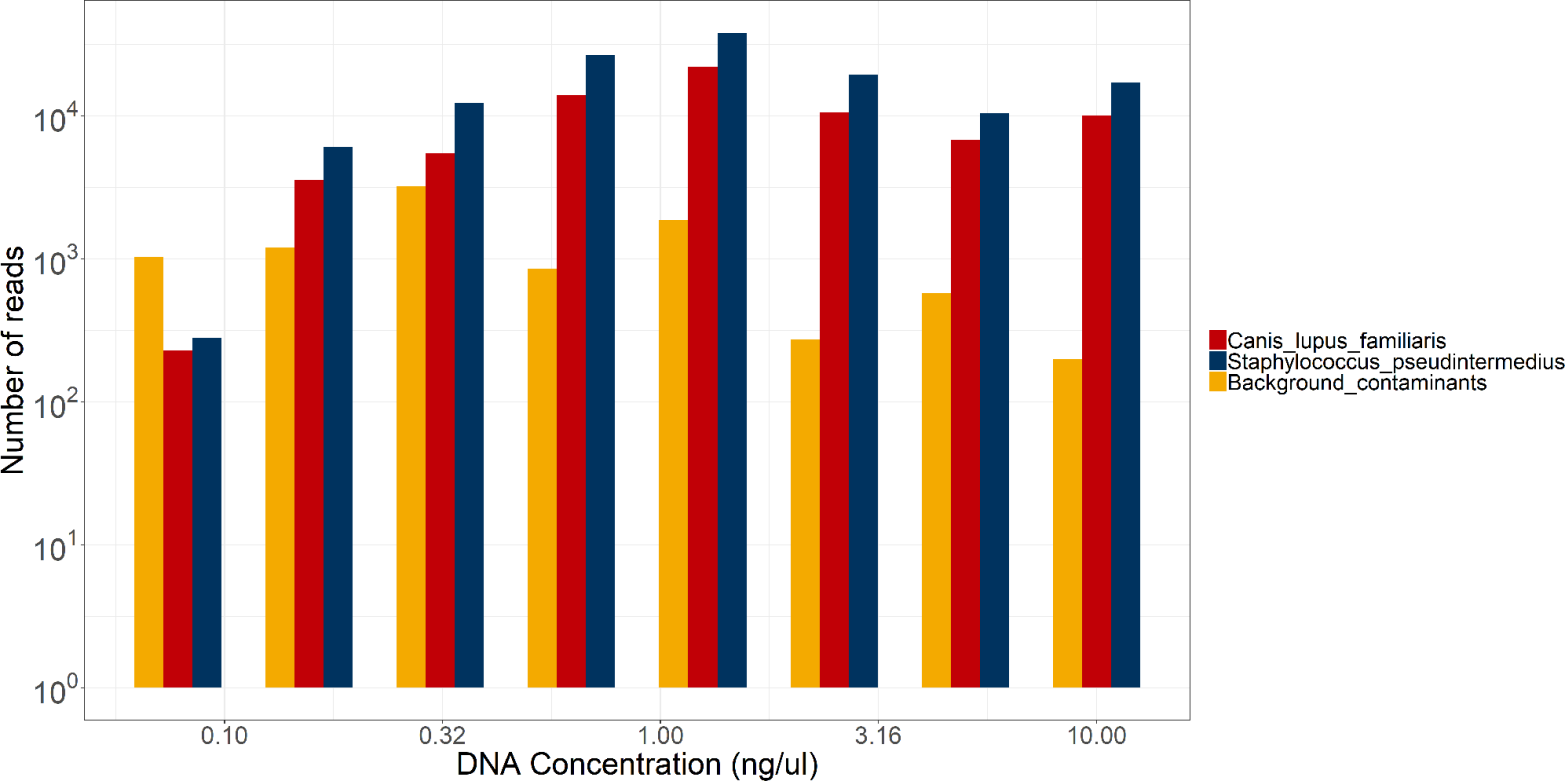
Sequencing serial dilutions of *S. pseudintermedius* and *C. lupus familiaris* DNA1:1 dilution of gDNA in nuclease-free water, sequenced for 24 hours using SQK-RBK004 (rapid barcoding) and R9.4.1 MinION flow cells. A) shows the number of reads identified by EPI2ME’s WIMP tool as the correct species vs starting gDNA concentration. B), C) and D) show the number of reads identified as the correct species vs the number identified as the next most common species for *E. coli *, *S. pseudintermedius* and *S. canis *, respectively.

A small number of *S. pseudintermedius* and *C. lupus familiaris* reads were seen in our negative control (12 and 8 reads, respectively). These represent barcode crosstalk between different samples; the spillage of barcode from a more highly concentrated sample into a less highly concentrated (that is, negative) sample. The level of barcode crosstalk seen here was, however, extremely low, especially compared to the number of background contaminant reads seen in the negative control. Around 2000 contaminant reads were seen in the negative control after 24 h of sequencing; these likely came from the kits and tubes used throughout DNA extraction and library preparation, and were identified as ‘*Homo sapiens’* and ‘* Nocardioides alcanivorans *’ by Kraken2 with our bacteria_plus database (see Results SR1 for further discussion of our experiences with this database, including the identification of contaminants). After subtracting the crosstalk numbers from the other test samples, we found that the *S. pseudintermedius* DNA was still identifiable at concentrations much lower than our means of 4.86 ng µl^−1^ (skin swabs) and 21.98 ng µl^−1^ (urine). As seen in Fig. 2, both *S. pseudintermedius* and *C. lupus familiaris* were seen at levels above background contamination with starting concentrations as low as 0.16 ng µl^−1^. In addition, even at 0.08 ng µl^−1^, where the *S. pseudintermedius* reads were outnumbered by the contaminants, *S. pseudintermedius* reads were still seen at an abundance far greater than 1%, hence would be called as a positive (especially as the background contaminant reads would be identified as such and excluded by analysis of the negative control sample). Although we only tested once (due to cost limitations of MinION flow cells), we concluded that we could use the rapid barcoding kit to sequence even very low concentration clinical samples without the need for the PCR amplification step.

#### MinION flow cells produce sequencing data significantly faster than Flongle flow cells, and washing MinION flow cells allows their repeated re-use

The Flongle is an adapter which fits into MinION or GridION sequencers and allows the use of Flongle flow cells, which are a single-use, lower yield alternative to MinION flow cells. These characteristics of Flongle flow cells are desirable for clinical use: single-use means no potential cross-contamination between different samples, and the lower yield means the flow cells are correspondingly less expensive than MinION flow cells. Clinical applications for which Flongle flow cells are already being used include rapid sequencing of viruses such as SARS-CoV-2 and monkeypox [65, 66], HLA-typing [67, 68], and 16S metagenomics [7, 69]. A number of previous studies have also investigated the use of Flongle flow cells for the rapid identification and typing of bacterial infections in some sample types [14, 25, 70, 71]. For veterinary purposes, where even in a hospital sample numbers may be low, sequencing single samples presents a more practical and cost-effective solution than either waiting for enough samples for a full MinION flow cell run or using a whole MinION flow cell per sample.

The major downside of Flongle flow cells with regards to rapid sequencing is their reduced number of pores; with up to 126 pores available on a Flongle flow cell compared to up to 512 available at any one time to sequence on a MinION flow cell, DNA is sequenced correspondingly less rapidly on a Flongle flow cell. However, when sequencing low concentrations of DNA, pore occupancy is often low on a MinION flow cell (not every pore is sequencing and many are empty because of the low number of DNA strands loaded), hence it was possible that sequencing speeds would be similar on both types of flow cell.

We therefore sought to evaluate the utility of Flongle flow cells in our protocol compared to the MinION, sequencing two clinical samples on each, and comparing the length of time taken to produce the same volume of data on each (Table S4). The volume of DNA sequence data produced by Flongle in a 24 h period was matched by MinION within 1.5 h for one sample, and 3 h for the other, even for relatively low masses of loaded DNA. The MinION therefore produces data much faster than the Flongle, even for low concentration samples.

However, as mentioned above, although MinION flow cells offer greater sequencing speed, the price of each MinION flow cell precludes the use of one flow cell per sample. Barcoding with SQK-RBK004 allows multiplexing of up to 12 samples, whilst barcoding with more recent kits allows multiplexing of up to 96 (SQK-RBK110.96), but in a clinical setting there may not be sufficient samples to load a full 12 + sample flow cell and still produce results within the desired timeframe.

We therefore examined the possibility of re-using MinION flow cells. This would involve sequencing individual samples for as long as necessary to produce the sequence data required, stopping the run and performing a DNase wash of the flow cell, then storing the flow cell until another sample was received. In this way, the same flow cell could theoretically be used many times, thus reducing the cost-per-sample without the need for simultaneous sequencing of multiple samples. Accordingly, we aimed to establish i) how many times could we re-use a flow cell to produce sufficient sequence data in a timely manner, and ii) how much residual DNA from previous samples would remain in the flow cell after washing. Employing a cultured *E. coli* sample, we examined the capacity to repeatedly produce 200 Mbp of DNA within 2 h, and what percentage of the reads had the correct barcode attached for each post-wash sequencing run.

**Fig. 3.**
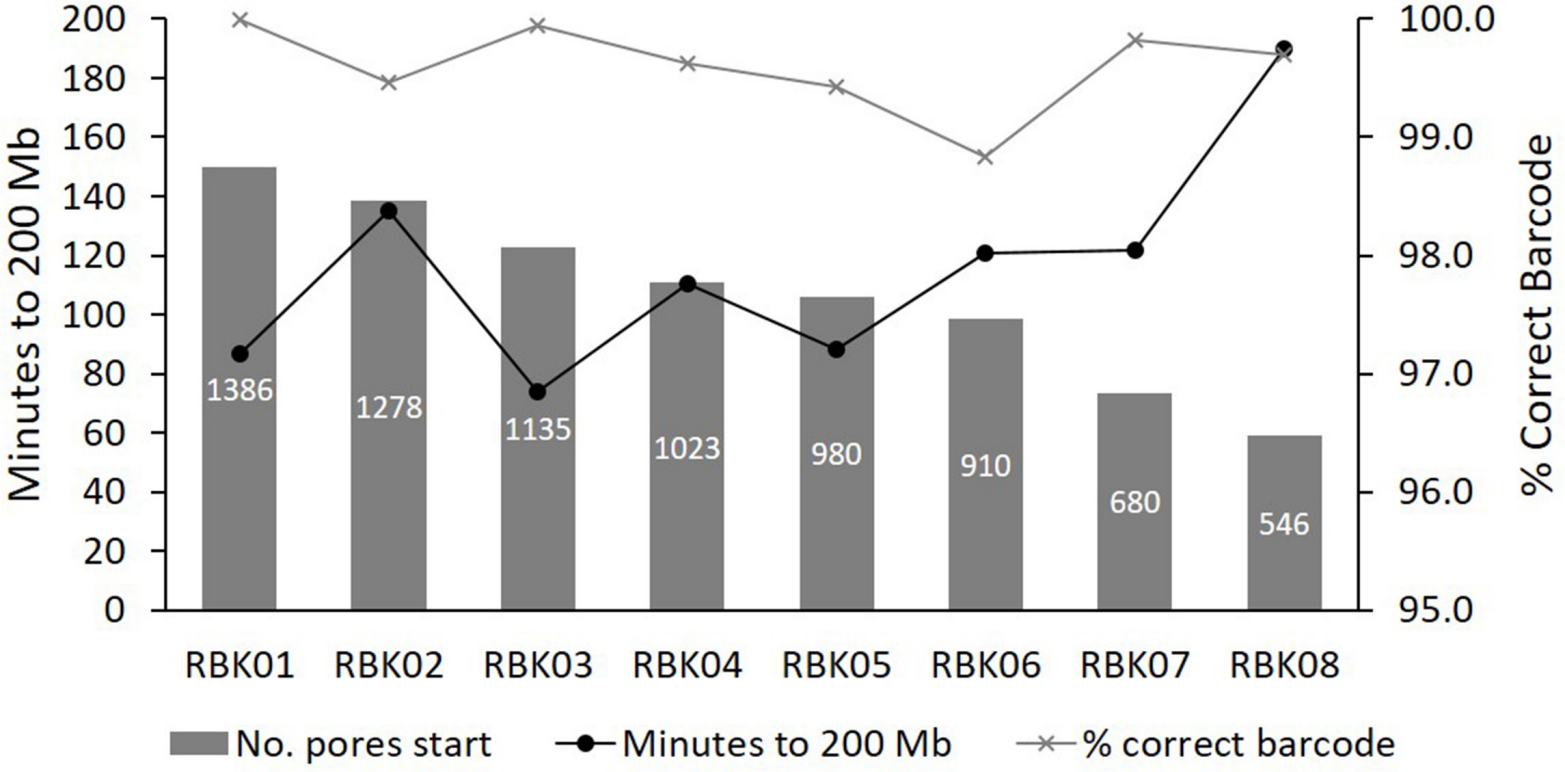
MinION flow cell progression when washing and re-using multiple times. The same 108 ng µl^-1^ *E. coli* sample was sequenced up to 200Mbp in eight consecutive sequencing runs on a single flow cell. In between runs, the sequencing was ceased, and the flow cell was washed with wash kit EXP-WSH004-XL according to manufacturer’s instructions. The flow cell was either then stored over one or two nights with storage buffer, or the next sequencing run was immediately commenced.

We were able to use the same flow cell eight times before it was exhausted, and the percentage of reads with the correct barcode never fell below 98.8 % (Fig. 3, Table S5). Although using different barcodes for each subsequent sample reduces the risk of cross-contamination between runs to negligible, especially when paired with a negative control sample each time to control for barcode misclassification, the low levels of DNA carry-over means that available pores were, in general, not being used to sequence previous samples, which would be inefficient and waste time. We also later postulated that only 100 Mbp of sequence may be sufficient for species identification and AMR prediction (representing up to 20 x coverage of the average 5 Mbp bacterial genome [72], dependent on levels of host contamination), hence flow cells could potentially be used even more times.

Since the quality and longevity of the pore array likely varies from flow cell to flow cell, and likely also depend on the nature of the samples being sequenced, we also trialled a second flow cell, using real clinical urine samples that had been extracted on the same day they were sequenced (Table S7). This second flow cell was able to produce 100 Mbp each for nine samples before being used to exhaustion, with samples being sequenced in batches of one to four per run, and over a period of roughly 1 month. Ultimately, we estimate that the average MinION flow cell can be reliably used and re-used at least eight times to sequence 100–200 Mbp per sample. The slight increase in cost of re-using MinION flow cells versus Flongle flow cells is balanced against producing the data at least eight times faster than it would be on a single-use Flongle flow cell, as well as producing less plastic waste (especially as MinION flow cells can be returned to ONT for recycling, whilst Flongle flow cells cannot).

### Testing the optimised extraction and sequencing process on real clinical samples

Having established an extraction and sequencing protocol, and a strategy for using and re-using MinION flow cells, we next wanted to test the performance of the finalised protocol for processing clinical samples in real-time, measuring the time taken from sample to result as well as establishing the accuracy of our results compared to those produced by the current gold-standard culture-based tests.

#### A wide range of DNA concentrations were retrieved from clinical samples, and we were able to extract DNA from all but the lowest abundance samples

During the development of this protocol, 44 clinical urine and skin swab samples (19 urine and 25 skin swab) were processed. Of these, 35 produced enough DNA to be sequenced, using either the rapid barcoding or rapid PCR barcoding library preparation kit (see library kit, Guppy version and MinKNOW version in Table S2), and either MinION or Flongle flow cells. These samples were not usually processed on the same day they were received; instead, they were processed and sequenced in batches, and the raw sample and/or extracted DNA was stored at 4 °C in the meantime. After finalising the protocol, we processed a further nine ‘post-development’ urine samples in real-time, which involved collecting, processing and producing results all on the same day. This equalled a total of 53 clinical samples processed here (44 during development and nine post-development), of which 43 were sequenced (35 development and eight post-development) (Tables 4, S2, S6 and S7).

**Table 4.** Results from processing nine clinical urine samples in real-time using our final optimised protocol

Sample name	Processing time to sequencing (hours)	Processing time to full results (hours)	Species Kraken	Species VITEK 2	AMR sequencing	AST VITEK 2
**Post-dev-1**	5	8	* S. aureus *	* S. aureus *	Ampicillin/Penicillin, Tetracycline, Fosfomycin, Tigecycline	Penicillin
**Post-dev-2**	04 : 35	05 : 30	* E. coli *	* E. coli *	Cephalosporins, Colistin	Cefalexin, Cefalotin
**Post-dev-3**	05 : 15	8	* E. coli * * Streptococcus gallolyticus *	*E. coli [2]* *Streptococcus gallolyticus ssp pasteurianus*	Trimethoprim, Tetracycline	Cefalexin, Cefalotin Cefalexin, Cefalotin (I)
**Post-dev-4**	05 : 15	8	na	No growth	None	na
**Post-dev-5**	4	4	na	No growth	na	na
**Post-dev-6**	05 : 15	8	* Mycoplasmopsis canis *	No growth	None	na
**Post-dev-7**	5	8	* E. coli *	*E. coli [2]*	Cephalosporins, Class A beta-lactams, Colistin	Ampicillin, Cefalexin, Cefalotin, Chloramphenicol (I)
**Post-dev-8**	5	8	* Proteus mirabilis *	* Proteus mirabilis *	Trimethoprim, Chloramphenicol, Tetracycline, Streptomycin, Sulfonamide, Class A beta-lactams, Streptothricin, Spectinomycin	Ampicillin, (I) Co-amoxiclav, (I) Cefalotin, Enrofloxacin, Marbofloxacin, Predofloxacin, Doxycycline, Tetracycline, Nitrofurantoin, Chloramphenicol, Trimethoprim/Sulfamethoxazole
**Post-dev-9**	5	8	* E. coli *	* E. coli *	Cephalosporins, Class A beta-lactams, Colistin, Class C beta-lactams, Streptomycin, Streptothricin, Trimethoprim	Ampicillin, Co-amoxiclav, Cefalexin, Cefalotin, Chloramphenicol (I)

A wide variety of DNA concentrations were retrieved from these samples, ranging from 0 to >120 ng µl^−1^ for the urine samples (mean=37.57, SD= 48.23) and 0 to 116 ng µl^−1^ for the skin swabs (mean=22.51, SD=32.45). As mentioned previously, the lower limit of detection for sequencing was determined to be < 1 ng µl^−1^ of cleaned-up DNA. In total, only four urine samples and five skin swabs produced DNA with lower concentrations than our lower limit, and colony forming units (c.f.u.) per millilitre of sample indicated that those samples had either no growth of any kind, or contained fewer than 1×10^5^ cells. We therefore estimate our lower limit for sufficient DNA from clinical samples to be between 1×10^5^ and 1×10^6^ c.f.u. ml^−1^. The exact lower limit is likely to depend on other sample characteristics, such as the presence of host cells, sediment or inhibitors; some of these factors may assist DNA extraction, whilst others may be detrimental.

#### Our optimised protocol can accurately identify species within five hours

In order to test relevance to a clinical scenario, nine post-development urine samples were processed in real-time. Quantifiable levels of DNA were extracted from eight samples; the remaining sample was classified as ‘negative’ after the DNA extraction, at roughly 4 h after the sample was received. The average time taken to process the remaining eight samples through to the commencement of sequencing was just under 5 h (Tables 4 and S7). Using the online EPI2ME tool, species identification was carried out in real-time as soon as the first sequencing reads were produced. The speed of EPI2ME can be affected by the number of concurrent online users, but we were able to produce preliminary species identification calls within approximately 20 min of sequencing. In each case, we continued to sequence until 100 Mbp of reads had been produced, theoretically representing up to 20 x coverage of the average bacterial genome, depending on levels of host contamination.

EPI2ME uses a database which includes species for which reference exitsts NCBI RefSeq database for viruses, bacteria, fungi and archaea, and does not include *C. lupus familiaris*. The tool can be prone to misclassifying missing species as either ‘*Homo sapiens’* or ‘* Escherichia coli *’ if they are not present in its standard database. To reduce the likelihood of false positive calls, we therefore also performed species identification using Kraken2 with two custom databases after sequencing was complete. The ‘pathogens_plus’ database is smaller and therefore produces results more quickly, but could misclassify species if the pathogen being sequenced was missing from the database. Hence, we also ran Kraken2 with the ‘bacteria_plus’ database, which contains all RefSeq representative bacterial genomes and therefore can clarify any ambiguous results from ‘pathogens_plus’. Notably, for our post-development samples, the initial rapid EPI2ME species calls differed from the Kraken2 calls for only one sample, in which EPI2ME detected both *P. mirabilis* and *E. coli *, whilst Kraken2 only identified *P. mirabilis* at an abundance greater than 1 % of the bacterial reads, a commonly used threshold for accepting true positive results [18, 73]. The culture-based diagnostic process also only identified *P. mirabilis *, indicating a false positive result from EPI2ME. Across all the clinical samples processed during the study, EPI2ME and Kraken2 agreed on the species present in all samples except this one, and one other (SkSw08A, where EPI2ME detected *Finegoldia magna* in addition to *E. coli* and *E. faecalis *, but Kraken2 did not, Table S2.

Culture-based VITEK two results were also available for 32 samples (23 development samples and the nine post-development samples). These were in almost 100 % agreement with both EPI2ME and Kraken2 (Tables 4 and S7). For two development samples (SkSk08A and SkSw10) EPI2ME identified additional species compared to the VITEK two results (* Finegoldia magna* and *Escherichia coli *, respectively). As mentioned above, with our Kraken2 databases, *Finegoldia magna* did not occur at a greater than 1 % abundance, thus was deemed to be a (non-pathogenic) member of the background skin microbiome. Likewise, interpreting the results of our Kraken2 SkSw10 results alongside our negative control revealed the ‘* Escherichia coli *’ identified by EPI2ME was actually background contamination. This demonstrates the value of running additional thorough taxonomic classification with more extensive databases after EPI2ME real-time analysis, as well as the necessity of negative controls for every extraction and sequencing run.

We were able to extract DNA from two post-development urine samples for which no growth was seen via culture (post-dev-4 and post-dev-6). On species identification with Kraken2, the DNA from one (post-dev-4) was found to be due to contamination, and therefore a false positive. The DNA extracted from post-dev-6, however, was shown by both EPI2ME and Kraken2 to be from *Mycoplasmopsis canis. Mycoplasma* and *Mycoplasmopsis* species are notoriously fastidious and difficult to culture [74]; therefore, we believe that in this case, it is highly likely that the sequence-based approach was more sensitive than the current gold-standard culture-based techniques. It is worth noting that while culture-based species identification and ASTs are the ‘gold standard’ they are themselves not 100 % accurate, with problems including small sample sizes compared to the infecting population, results skewed to easily cultured and faster growing organisms, and laboratory error (e.g. single doubling dilution steps between sensitive, intermediate and resistant). A 2007 evaluation estimated VITEK two accuracy for species identification to be 98.3%, while AST accuracy was estimated to be 97.7 % [75].

#### Metagenome-based AMR prediction can be highly accurate, but is dependent on bacterial sequence data volume

The principle of predicting AMR phenotypes from sequencing data alone involves screening the data for known AMR-related genes or SNPs, based on curated databases like ResFinder or CARD [76, 77]. The accuracy of using sequencing data alone for AMR identification varies from species to species, but previous studies have suggested >97 % accuracy for *E. coli* and over 99 % for *Staphylococcus aureus* [78, 79]. This level of accuracy is, however, dependent on the volume of sequencing data available, the complexity of the samples being sequenced, and the amount of contaminating host DNA present. Here we interrogated DNA sequence produced from the 32 urine samples and skin swabs with paired VITEK two results, using our metagenomic protocol for AMR detection and comparing the results to the phenotypic data produced by VITEK two AST, looking at combined ‘Resistant’ and ‘Intermediate’ phenotypes as well as ‘Resistant’ alone (Tables 4, S2, S6 and S7).

For urine samples, 71.7 % of the combined ‘Resistant’ and ‘Intermediate’ resistance phenotypes identified by phenotypic AST were also predicted by our protocol. We found 50 % of samples were in exact agreement for all AMR calls for each sample. Further investigation revealed that the vast majority of AST calls that did not correlate with the sequence-based predictions were defined as intermediate resistance, often to chloramphenicol, which may represent unknown genetic mechanisms. Excluding intermediate resistance calls from the VITEK two results (i.e. looking only at the ‘Resistant’ calls), we detected 83.7 % of the same AMR using sequencing data alone. Excluding a single missed gene (likely an *oqx* gene conferring resistance to multiple antimicrobials) in one of our more complex post-optimisation samples (post-dev-8) increases the sensitivity of our AMR predictions to 95.3 % (41 of 43 resistant phenotypes accurately predicted, excluding intermediates).

The remaining two AMR phenotypes not detected by our pipeline were from one sample (post-dev-3), which was determined by the VITEK two to be a co-infection consisting of two different strains of *E. coli* and one strain of *Streptococcus gallolyticus* spp. *pasteurianus *. Though the type of resistance (multiple cephalosporins) identified in this sample was frequently detected accurately by our pipeline, the complexity of this sample may have hindered our AMR prediction. The selection of 100 Mbp as a target was based on a compromise between genome coverage and sequencing time; 100 Mbp represents 20 x coverage of the average bacterial genome [72] and is usually easily sequenced in under 2 h. However, we propose that complicated co-infection samples may require significantly more than 100 Mbp of sequencing data, particularly when host DNA is also present (Kraken2 identified 47.5 % of the DNA in this sample as canine). A potential solution for this is the recently improved EPI2ME Labs software, which allows the real-time use of tools such as Kraken2 (with or without custom databases) whilst data is still being sequenced. Real-time analysis will indicate the complexity of a sample, as well as the levels of host contamination, thereby allowing a more informed decision about when ‘enough’ data has been produced. In the meantime, our pipeline can predict the vast majority of resistant phenotypes in non-complex urine samples.

In contrast, for skin swab samples, of which five had paired VITEK two phenotypes, effective sequence-based prediction of AMR was not possible due to the low abundance of the bacteria in the samples, (3×10^2^ to 7.5×10^5^ c.f.u. ml^−1^), almost all below our predicted lower limit of detection. In addition, four of the five samples were more than 95 % dog DNA, so bacterial DNA comprised a very low proportion of the extracted DNA (reflecting one or two times coverage). Although species can still easily and quickly be identified for these low abundance skin samples from one or two times coverage, the amount of sequencing data required to accurately detect AMR genotypes is likely cost-prohibitive for veterinary applications, as it would reduce the number of uses of each flow cell. One of our urine samples (post-dev-9) contained over 83 % dog DNA, as well as a multidrug-resistant *E. coli *, yet we were able to predict all four of its non-intermediate AMR phenotypes from just 100 Mbp. This suggests that even small increases in the relative levels of bacterial cells we are seeing in our low abundance skin swabs could greatly improve our ability to accurately detect AMR genotypes.

Subsequent to sequencing our clinical samples, we tested a feature of the GridION, adaptive sampling, which allows the rejection of DNA which maps to a reference genome during sequencing, and which can therefore potentially deplete some of the dog DNA seen in our samples. We have included our results in Supplementary Results SR2*,* which shows that adaptive sampling did greatly reduce the proportion of dog reads sequenced compared to bacterial. Nonetheless, removing host DNA during sequencing is costly in terms of time, as pore-time is still wasted sequencing host DNA until it is rejected, and the repeated rejection of host DNA by reversal of current across individual pores may reduce the overall life-span of a flow cell, resulting in fewer re-uses and therefore increased per-sample cost. In addition, adaptive sampling requires a powerful computer (or GridION) to map reads and make decisions in real-time, the cost of which may preclude its use in smaller clinics. Therefore, reducing the amount of host DNA present in a sample prior to sequencing is likely a better a strategy. Previous studies have shown promising results for pre-extraction host depletion using a range of techniques including saponin-and-DNase enzymatic methods, PMA plus UV-light-based chemical methods, and even methods as simple and cost-effective as physical filtering of host cells through a 22 µm filter prior to extraction [18, 80, 81]. A kit also exists (New England Biolab’s NEBNext Microbiome Enrichment kit) which can reduce the levels of eukaryotic DNA in a sample after extraction, based on the predicted higher levels of methylation on eukaryotic DNA compared to bacterial. These methods will need to be explored further, and in the future probably incorporated into our protocol for all sample types which are likely to contain high levels of host cells (such as skin, blood and tissue).

#### Concluding comments and future considerations

We have developed and validated a protocol for the rapid, culture-free, agnostic identification of pathogenic species from clinical canine urine and skin swab samples, by cost-effective metagenomic sequencing. We have shown that this protocol is capable of detecting a wide array of species, representing over 90 % of the urine and skin infections seen in the R(D)SVS HfSA. Although we did not test the remaining 10 % of species due to their large number and relative rarity, our metagenomic extraction should effectively extract DNA from any species present in a sample. Further, we have shown that we can identify the same species identified by culture-based testing in 100 % of the samples we sequenced, using sequencing data alone. False positive results can be ruled out by the use of negative controls and multiple different taxonomic classification methods, and we have also shown an ability to detect pathogenic species which are generally undetectable by culture. A small number of samples had pathogens which were detected by culture but for which we were unable to extract DNA, but these had CFUs ml^−1^ counts of 10^5^ or less, indicating a lower limit of detection for our protocol of between 10^5^ and 10^6^ CFUs ml^−1^. We intentionally developed a protocol that can also be adapted to a variety of other sample types, and the MagAttract protocol can be easily adapted for tissue, blood and other bodily fluids. In this way, one simple protocol can be deployed in a clinical setting to detect pathogens in a wide variety of infections, in different animals, in as little as 5 h, compared to the 48 h plus commonly seen in the current gold-standard diagnostics techniques.

Although the large amounts of host cell contamination we saw in skin swab samples was problematic with regards to the prediction of AMR, a number of depletion techniques exist which could be incorporated into our protocol, at the cost of time and money, but with the benefit of allowing accurate AMR prediction from a greater range of samples. Besides the samples with the highest levels of host DNA contamination, our ability to match ‘Resistant’ VITEK two calls was relatively accurate, approaching 95 % in all but the most complicated co-infections. However, a number of samples displayed ‘Intermediate’ VITEK two resistance to certain antimicrobials, mainly chloramphenicol, which were not predicted from the sequencing data. This suggests that as-yet unknown mechanisms may be responsible for intermediate resistance, or that certain mechanisms are currently missing from the AMR databases used (NCBI, ResFinder and CARD). Our current AMR prediction pipeline includes combining the results of three different tools (EPI2ME, Abricate and AMRFinderPlus), which function in different ways, in order to capture all potential information from our sequencing data.

The methods validated herein focus primarily on the wet-lab; we have used bioinformatics tools which have already been previously well-described and well-validated, including for use with ONT long reads and in human and veterinary clinical metagenomics, and we did not seek here to benchmark them in any further detail than already available in the peer-reviewed literature [18, 32, 73, 82–86]. Nonetheless, future optimisations of our pipeline will likely include testing alternative tools and databases, and producing a finalised easy-to-run data analysis workflow. Potential alterations to the methods used here include the addition of Bracken after Kraken2 for species identification, as a recent paper showed this approach to be more accurate and less prone to misclassification of clinical metagenomic samples than Kraken2 alone using long reads [84]. Likewise, our current AMR prediction tools may be replaced by the newly released, ISO-certified, abritAMR, which combines the approaches of the tools used herein [87]. Future developments to the wet-lab aspects of our protocol include, as described above, the potential inclusion of host depletion steps prior to sequencing. We also note that relatively few of the samples we tested here represented co-infections, although we were able to identify those that did (for example, sample post-dev-3). Future testing of larger numbers of clinical samples will help to validate our ability to accurately identify all pathogens in co-infection samples.

Although we focussed on urine and skin swab samples here, we aimed to design a protocol which could be adapted to an array of other sample types (e.g. blood) from infections in other animals including humans. Moreover, the approach could be used in a variety of clinical settings, from small practices to large hospitals. By comparing and optimising a number of different kit-based gDNA extractions and sequencing techniques, combined with community-built DNA analysis tools, we have developed a pipeline which can identify the bacterial species present in clinical samples in as little as 5 h. It can also predict the antimicrobial resistance phenotype of those species with up to 95 % accuracy, in around 8 h.

Lastly, we note that the flow cells (R9.4.1) and kits (SQK-RBK004) used here throughout development are now being replaced with R10.4.1 and SQK-RBK114. We expect these new flow cells and kits to incorporate seamlessly into our existing protocol and, indeed, they will likely improve the accuracy of the sequencing data produced, which could in turn improve the accuracy of our species identification and prediction of SNP-based AMR.

## Supplementary Data

Supplementary material 1

Supplementary material 2
