# Supplementary material for: Rapid metagenomic sequencing for diagnosis and antimicrobial sensitivity prediction of canine bacterial infections
Source: Microb Genom. 2023 Jul 20;9(7):mgen001066. doi: 10.1099/mgen.0.001066 (PMC10438823; doi:10.1099/mgen.0.001066)
Supplement: Supplementary material 2 [file mgen-9-1066-s002.pdf]

Supplementary Material: Rapid metagenomic sequencing for diagnosis and antimicrobial sensitivity prediction of canine bacterial infections

Supplementary Figures

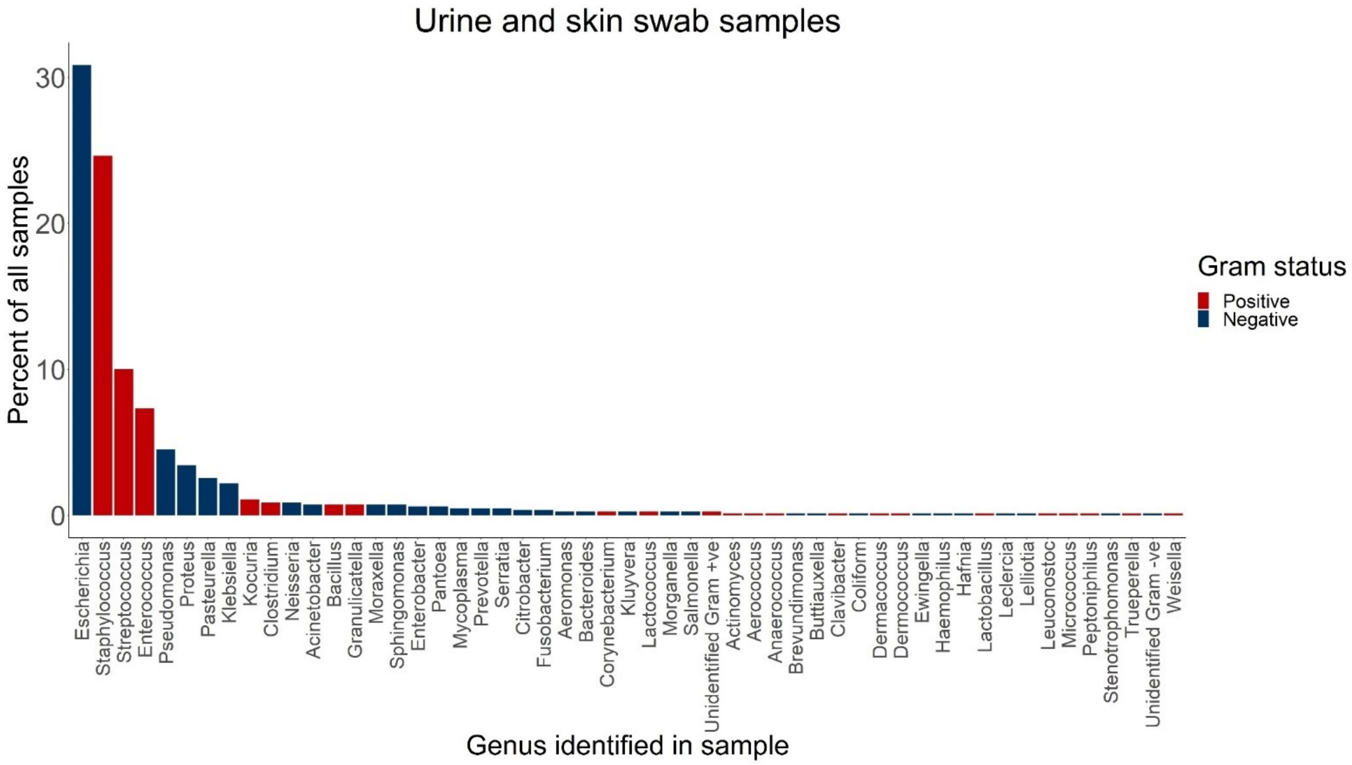

Fig. S1 Pathogens identified in HfSA urine and skin swab samples, 2018 & 2019.
Blue text indicates Gram –ve species, red text indicates Gram +ve species. There was roughly a 50:50 split (52% Gram –ve, 48% Gram-ve)

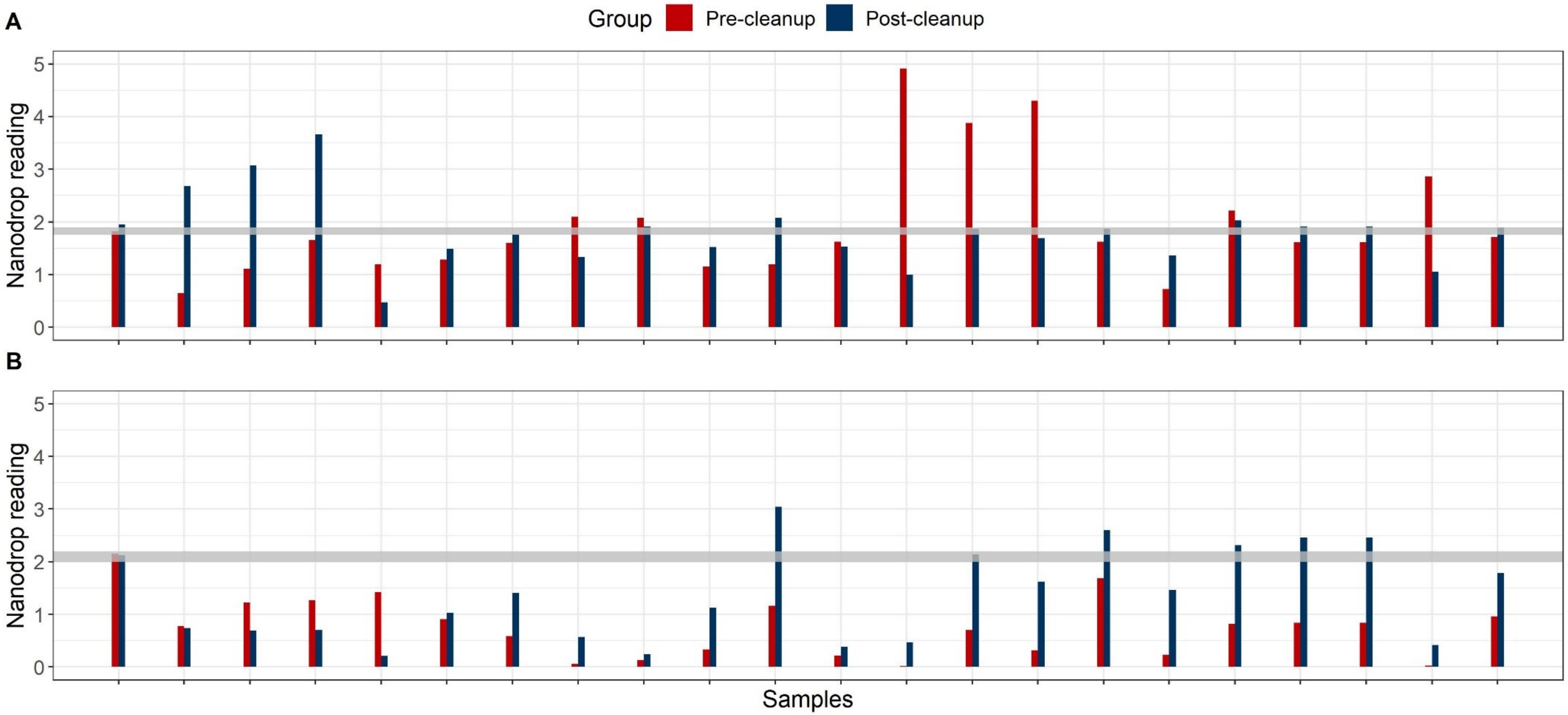

Fig. S2 Nanodrop 260/280 (A) and 260/230 (B) ratios for 22 clinical samples, measured before and after ProNex bead clean-up.
The dark grey rectangles on each plot indicate the ideal ranges for each ratio.

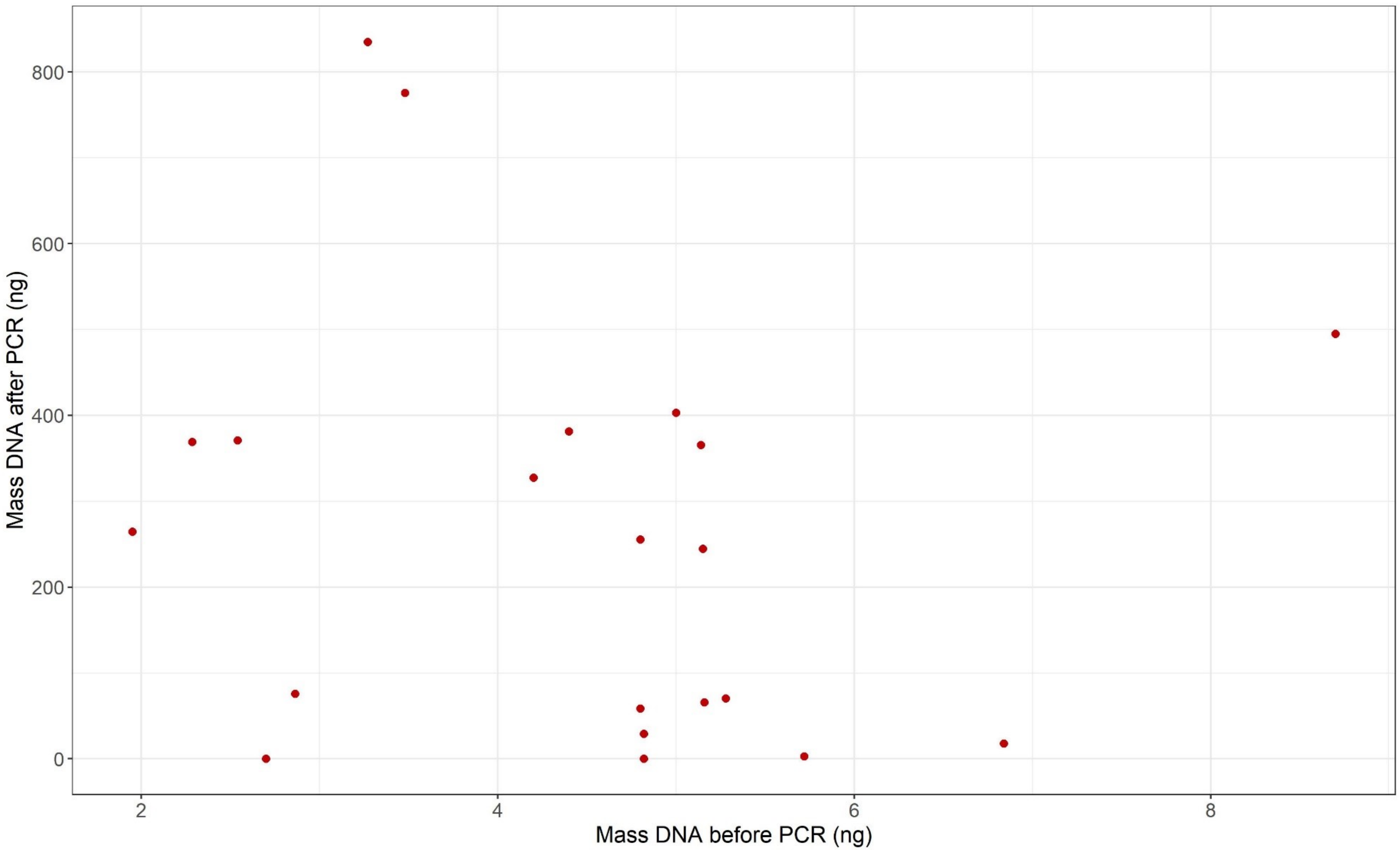

Fig. S3 Inconsistent efficiency of the PCR in the Rapid PCR Barcoding (SQK-RPB004) library preparation kit.
21 samples of varied starting concentration (hence varied mass of DNA used in PCR reaction) were amplified by the SQK-RPB004 PCR reaction, and their amplified DNA concentration in 10 µl was measured by the Qubit HS DNA kit. Extremely limited correlation was seen between pre- and post-PCR DNA mass, suggesting inconsistency of the PCR reaction.

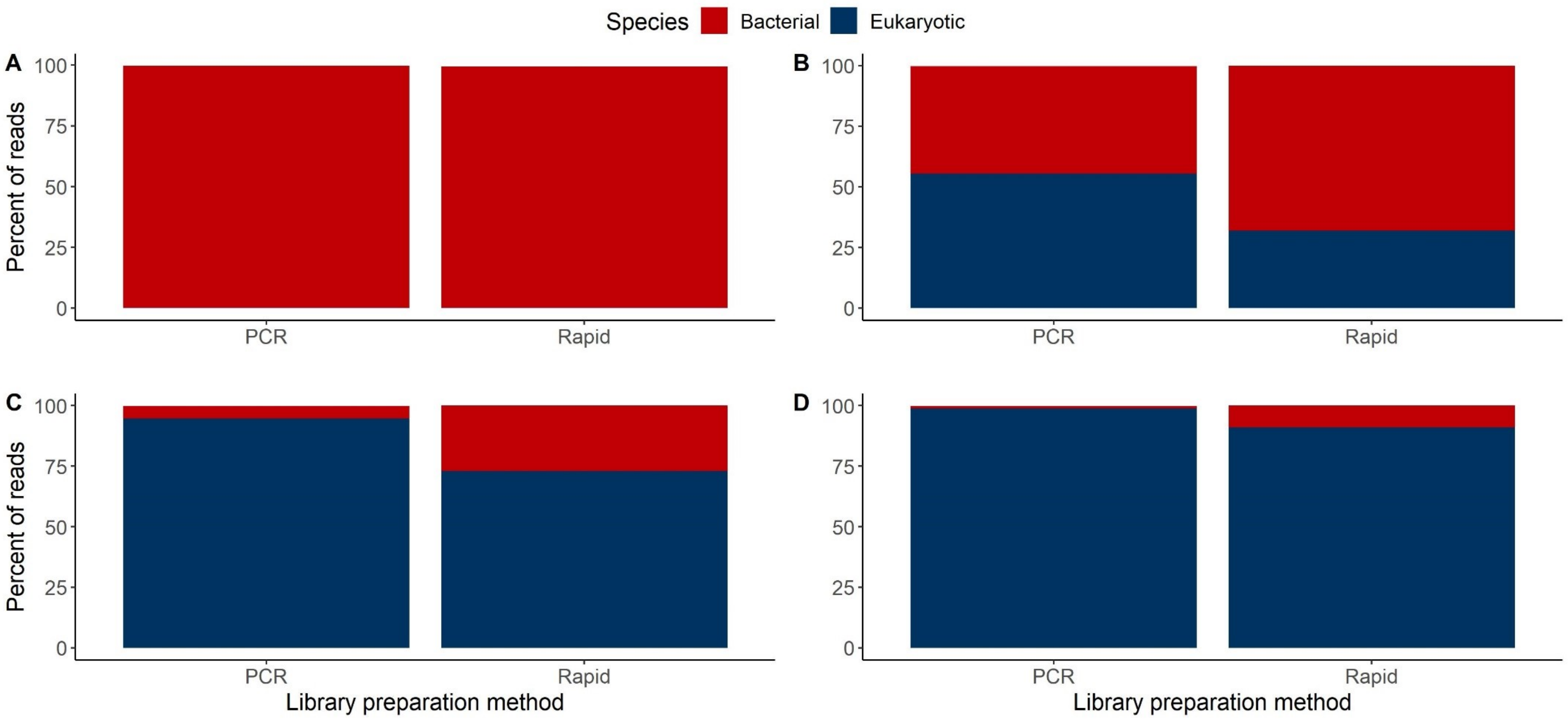

Fig. S4 Selective eukaryotic DNA amplification by PCR.
Four clinical samples were sequenced with both the Rapid PCR Barcoding (SQK-RPB004) and Rapid Barcoding (SQK-RBK004) kit for 24 hours on R9.4.1 MinION flow cells. A) and B) represent urine samples DTU09 and DTU16 respectively, while C) and D) skin swab samples SkSw08A and SkSw14 respectively.

Supplementary Results SR1

Testing of Kraken2 with custom databases pathogens_plus and bacteria_plus

Background

Kraken2 is a *k*-mer-based taxonomic classification tool commonly used in metagenomic studies (1, 2). Whilst pre-built databases exist, custom-built databases are also frequently used (3). For our purposes, identifying pathogens in clinical veterinary samples which may contain high levels of host DNA, we required a database which could classify reads from bacteria as well as a number of animal species. Therefore none of the pre-built, pre-tested databases suited our need, and we built two custom databases. The first, pathogens_plus, contains 668 genomes belonging to known bacterial, viral, protozoan and fungal pathogenic species, including those we found in a 2014 study listing the top 100 human and animal pathogens in Europe (4) (we could not find similar lists at the time of database construction for other parts of the world). The second, bacteria_plus, contains 16,113; all of the genomes classed as "representative" or "reference" in the NCBI RefSeq database in November 2022. Both custom databases also include eight selected mammalian genomes known to be of interest for our current or future research (*Canis lupus familiaris*, *Homo sapiens*, *Felis catus*, *Equus caballus*, *Oryctolagus cuniculus*, *Sus domesticus*, *Bos taurus* and *Ursus arctos*).

A recent benchmarking study (5) tested taxonomic classification tools using both Illumina and Nanopore reads, with common blood-stream pathogens (including several of the top 10 species seen in canine urine and skin infections) and blood-culture contaminants. Kraken2, used with the standard 49 Gb database, containing all complete genomes from the RefSeq archaea, bacteria, viral, plasmid and human databases, was found to deliver the fastest classification speeds, with comparable accuracy to the other tools tested (Centrifuge and Bracken). Of note, Kraken2 classification followed by Bracken classification (as recommended by the authors of Kraken and Bracken, (6, 7)), using nanopore reads longer than 2,000 bp was found to produce classification accuracy on par with Illumina reads, whilst nanopore reads longer than 5,000 bp produced classification accuracy which outperformed Illumina reads.

The purpose of the additional work detailed here was to validate that both of our custom databases could detect, using R9.4.1 quality nanopore reads and with minimal risk for false positives and negatives, the species most frequently seen in our veterinary samples. A secondary aim was to determine the optimal confidence threshold to use with Kraken2 with our databases to minimise unclassified reads whilst also avoiding false positive classifications. Finally, we also tested a well-characterised two mock community (ZymoBIOMICS community standard and community standard log) as a proxy for an extremely (unrealistically) complicated co-infection.

Methods

Commands for tools mentioned are given on the project Github:
https://github.com/nataliering/Dogstails

Data sets

Two read sets were downloaded for the ZymoBIOMICS community standard, representing even and log distribution of the included organisms (**Table 1**). The read sets (accessions shown in **Table 2**) were downloaded using fasterq-dump from the SRA Toolkit (v3.0.0, https://github.com/ncbi/sra-tools) . These read sets were produced by Nicholls et al. (8), using R9.4.1 flow cells on a GridION, with Guppy (v2.2.2) basecalling (full Guppy config file: dna r9.4.1 450bps flipflop.cfg). Full experimental methods, including DNA extraction and library preparation, are given in the original study (8). 100 Mbp and 1 Gb randomly downsampled read sets were also produced for the even and log samples, using Rasusa (v0.6.1, 9).

Nine further read sets were downloaded, representing nine of the ten most commonly seen pathogens in urine and skin infections in dogs at the University of Edinburgh Hospital for Small Animals (HfSA) between 2018 and 2019 (see **Figure 1** in the main text). These read sets were selected by searching for the appropriate species on NCBI's SRA database, filtered by "Oxford Nanopore", followed by manual investigation of the resulting hits to find a read set with available sequencing metadata details (for example, flow cell version, basecaller details if possible). Where possible, the sample with the most similar sequencing metadata to our samples was selected (R9.4.1 flow cells, SQK-RBK004, GridION, Guppy v5+ in super accuracy mode). Where not possible, R9.4.1 flow cells were prioritised. For one species, *Proteus mirabilis*, no read sets with accompanying metadata were available.

Two of the ten most commonly seen pathogens, *Pasteurella canis* and *Kocuria rosea*, had no available Oxford Nanopore reads on the NCBI's SRA database. For this species, Badread (v0.2.0, 10) was used to generate 25x coverage of the *P. canis* genome in reads with a simulated accuracy close to that of Oxford Nanopore reads from 2020 kits and basecallers. The reads were simulated using the NCBI reference genome for each species.

NanoStat (v1.6.0, 11) was used to perform basic quality control for each read set. The fastq accessions for all read sets, number of reads and total number of bases, are shown in **Table 2**.

Testing Kraken2 with our custom databases

Each of the read sets described above was tested in full against both custom Kraken databases (bacteria_plus and pathogens_plus), at three confidence thresholds, 0 (default), 0.05 and 0.1

For the ZymoBIOMICS datasets, the Kraken2 the percent of reads assigned to each expected species were recorded. For the individually sequenced species datasets, the percent of reads assigned to the expected species, plus all other species >1%, were recorded.

Table 1: Species included in the ZymoBIOMICS community standards, and their expected proportions in the even and log distributions

Species	Type	Order	NRRL accession	Even expected proportion (%)	Log expected proportion (%)
Bacillus subtilis	Gram +	Bacillales	B-354	12	0.89
Cryptococcus neoformans	Yeast	Tremellales	Y-2534	2	0.00089
Enterococcus faecalis	Gram +	Lactobacillales	B-537	12	0.00089
Escherichia coli	Gram -	Enterobacterales	B-1109	12	0.089
Lactobacillus fermentum	Gram +	Lactobacillales	B-1840	12	0.0089
Listeria monocytogenes	Gram +	Bacillales	B-33116	12	89.1
Pseudomonas aeruginosa	Gram -	Pseudomonadales	B-3509	12	8.9
Saccharomyces cerevisiae	Yeast	Saccharomycetales	Y-567	2	0.89
Salmonella enterica	Gram -	Enterobacterales	B-4212	12	0.089
Staphylococcus aureus	Gram +	Bacillales	B-41012	12	0.000089

Adapted from Nicholls et al. (2019) (8)

Table 2: Datasets used here, and their characteristics

Dataset	Accession	Known sequencing metadata	Number of reads	Total bases (Mbp)	Quality (median Q)
ZymoBIOMICS even	ERR3152364	GridION, R9.4.1, Guppy flip-flop, v2.2.2, LSK-SQK109	3.49M	14,380	10.3
ZymoBIOMICS log	ERR3152366	GridION, R9.4.1, Guppy flip-flop, v2.2.2, LSK-SQK109	3.67M	16,510	9.8
Escherichia coli	DRR393831	GridION, R9.4.1, SQK-RBK004	28,568	202.0	13.6
Pseudomonas aeruginosa	SRR23473171	MinION, R9.4.1, SQK-LSK109, Guppy v6.1.5, SUP mode	95,937	155.8	14.9
Salmonella enterica	SRR22489994	MinION, R9.4.1. SQK-RBK004	81,007	521.2	10.1
Staphylococcus pseudintermedius	SRR16929174	MinION, R9.4.1, SQK-RBK004	78,755	311.4	14.5
Streptococcus canis	DRR218421	MinION, R9.4.1, SQK-RAD004	190,703	1,130	11.4
Enterococcus faecalis	SRR21979405	GridION, R9, SQK-LSK108	9,415	107.7	11.5
Proteus mirabilis	SRR14235433	Unknown	11,145	106.8	9.9
Pasteurella canis	NA	Reads simulated with Badread	11,623	57.5	11.4
Klebsiella pneumoniae	DRR393826	GridION, R9.4.1, SQK-RBK004	107,801	1,157	13.6
Kocuria rosea	NA	Reads simulated with Badread	20,488	98.8	11.4
Clostridium perfringens	ERR10360395	MinION, R9?, SQK-LSK108/9	37,694	83.3	11.0

Results and Discussion

Kraken2 with our custom databases can accurately identify all species in a complex microbial community

The aim of testing our custom databases (one pathogen-specific, and one pan-bacterial) with the ZymoBIOMICS microbial community standard was to determine whether each present species could be identified, despite the generally lower accuracy of nanopore reads produced with R9.4.1 flow cells. The ZymoBIOMICS microbial community standard contains eight bacterial species and two yeast species, and therefore represents a sample far more complex than any of the most complicated co-infections we would expect to see in a canine urinary tract or skin infection. Given recent work demonstrating that lower accuracy nanopore reads above 2,000 bp perform equally well as Illumina short reads for Kraken2 followed by Bracken with the standard databases (5), we were expecting successful species identification with our custom databases as well, although we do not currently use Bracken after Kraken2 classification for reasons of speed. As shown in **Table 3,** all species in the ZymoBIOMICS evenly distributed microbial community standard could be accurately identified by both of our custom databases, excluding the two yeast species are not included in bacteria_plus, whilst neither *Lactobacillus fermentum* nor *Saccharomyces cerevisiae* is currently included in pathogens_plus.

At the same time, we wanted to determine the optimal confidence threshold for nanopore reads with Kraken2, minimising both false positives and false negatives. The Kraken2 confidence threshold describes the proportion of kmers within each read which must map to a species before it is called as a true hit (0.05 is equivalent to 5% of the kmers within a read). The default confidence threshold if the user does not otherwise specify is 0; there is therefore a very high likelihood of reads being assigned to the wrong species. Little guidance is available regarding the best confidence threshold, and many studies using Kraken2 do not specify which threshold (if any) they used (3). Some benchmarking papers have found 0.05 to be an appropriate threshold for maximising precision/recall (12), whilst the authors of Kraken2 have indicated that the best threshold will vary according to context, but for general classification purposes 0.05 or 0.10 would suffice[1]. We therefore tested 0.00, 0.05 and 0.10 here. As seen in **Table 3**, as expected, at confidence 0.00 there was a tendency to overestimate the abundance of certain species, particularly *Bacillus subtilis*. At confidence 0.05, most of the species abundances predicted by Kraken2 with both of our databases were very similar to those found in the original study for which the data was produced (8), whilst at confidence 0.10, most species were underclassified. Of note, *Escherichia coli* was underclassified at all confidence thresholds except 0.00. This is a common problem with all taxonomic identification strategies, due to the extremely wide diversity of *E. coli* as a species, as well as its close similarity to other (currently) distinct genera, such as *Shigella* (13). In a database with only a single, or small number of, *E. coli* reference genomes, there is a low chance that the *E. coli* strain being sequenced is closely related to the reference(s), therefore many reads may be either misclassified (with a low confidence threshold) or unclassified (with a confidence threshold of 0.05 or above). This problem, which is seemingly unique to *Escherichia* and *Shigella* species, is discussed in further detail in the next section.

[1] "'Best' confidence threshold for general purpose classification?" Github community discussion: https://github.com/DerrickWood/kraken2/issues/167

Tables 3 and 4: Kraken2 results for the ZymoBIOMICS microbial community standard (even distribution) with our custom databases at three different confidence thresholds

**Even CS 100 Mbp**		**Confidence 0.00**		**Confidence 0.05**		**Confidence 0.10**	
**Species**	**Original %**	**Bacteria_plus %**	**Pathogens_plus %**	**Bacteria_plus %**	**Pathogens_plus %**	**Bacteria_plus %**	**Pathogens_plus %**
Pseudomonas aeruginosa	12	4.64	4.77	3.63	3.91	2.43	3.01
Escherichia coli	12	4.11	5.04	0.28	1.7	0.12	0.37
Salmonella enterica	12	6.06	6.17	4.62	5.17	2.85	4.11
Lactobacillus fermentum	12	13.58	Not in database	10.98	Not in database	8.52	Not in database
Enterococcus faecalis	12	10.57	10.52	9.14	9.07	7.64	7.61
Staphylococcus aureus	12	10.24	10.65	7.95	9.17	5.9	7.92
Listeria monocytogenes	12	12.41	12.62	3.48	11.08	6.08	9.56
Bacillus subtilis	12	18.17	16.66	12.04	9.08	9.64	3.05
Saccharomyces cerevisiae	2	Not in database	Not in database	Not in database	Not in database	Not in database	Not in database
Cryptococcus neoformans	2	Not in database	1.95	Not in database	1.09	Not in database	0.85
Unclassified	NA	10.5	26.24	27.04	44.71	38.37	57.48

**Even CS 15 Gb**		**Confidence 0.00**		**Confidence 0.05**		**Confidence 0.10**	
**Species**	**Original %**	**Bacteria_plus %**	**Pathogens_plus %**	**Bacteria_plus %**	**Pathogens_plus %**	**Bacteria_plus %**	**Pathogens_plus %**
Pseudomonas aeruginosa	12	4.84	4.94	3.71	3.67	2.56	3.11
Escherichia coli	12	4.16	5.17	0.27	0.12	0.09	0.39
Salmonella enterica	12	5.75	5.93	4.36	4.96	2.81	4.02
Lactobacillus fermentum	12	13.68	Not in database	11.06	Not in database	8.58	Not in database
Enterococcus faecalis	12	10.57	10.52	8.96	8.87	7.45	7.41
Staphylococcus aureus	12	10.26	10.7	7.98	9.22	5.9	7.9
Listeria monocytogenes	12	12.56	12.8	3.64	11.14	0.78	9.69
Bacillus subtilis	12	17.76	16.61	14	9.02	9.49	2.98
Saccharomyces cerevisiae	2	Not in database	Not in database	Not in database	Not in database	Not in database	Not in database
Cryptococcus neoformans	2	Not in database	1.93	Not in database	1.11	Not in database	0.87
Unclassified	NA	10.29	25.92	27.05	44.8	38.56	57.56

Next, we wanted to establish whether we could accurately estimate the species distribution within a complex microbial community from only small volumes of data; in our rapid sequencing protocol, we hope to be able to identify species and AMR from only 100 Mbp (though this will depend on levels of host contamination, as discussed in the main text). **Table 3** shows the Kraken2 results from a 100 Mbp randomly downsampled read set of the ZymoBIOMICS microbial community standard, whilst **Table 4** shows the same results from the full 15 Gb original dataset. Reassuringly, the species abundances predicted from the large and significantly downsampled read sets are strikingly similar: for the results at 0.05 confidence, a mean difference of 0.21% (±0.67, n=9) across all included species (including "unclassified") for the bacteria_plus database, and -0.23% (±0.52, n=9) for the pathogens_plus database. This result suggests that the use of a smaller dataset (100 Mbp, as planned for our rapid diagnostics protocol) should not be detrimental to the ability to detect species in any sample, including complicated co-infection communities.

Finally, we used the ZymoBIOMICS microbial community standard log distribution, in which the same ten species are found in different proportions, from 89.1% (*Listeria monocytogenes*) down to 0.000089% (*Staphylococcus aureus*) to estimate at what percentage our ability to detect a species would disappear. **Table 5** shows the results of running Kraken2 on 100 Mbp randomly downsampled from the original 17 Gb log distribution read set, with each of our custom databases and using our previously identified optimal confidence threshold, 0.05. We can see that using this confidence threshold is underclassifying most of the present species, but believe that underclassification is preferable to false positive classifications, especially in low abundance samples where false positives might be more likely (14). We can detect the presence of *Salmonella enterica* at 0.089% abundance with both databases, although we cannot detect *E. coli* at the same abundance for either database, for reasons already discussed. We cannot detect the species at the next logarithm step down (0.0089%, *Lactobacillus fermentum*) or anything smaller, suggesting that our limit of detection is around 0.089%. In any case, this exercise was largely academic, because we are applying a "true positive" detection threshold of 1% of microbial reads in our clinical samples, as it was determined by a number of previous similar clinical studies that this roughly equates to the threshold of what would be deemed a positive result by clinical culture (15, 16).

Table 5: Kraken2 results for the ZymoBIOMICS microbial community standard (log distribution) with our custom databases, at confidence threshold 0.05

**Log CS 100 Mbp**		**Confidence 0.05**	
**Species**	**Original %**	**Bacteria_plus %**	**Pathogens_plus %**
Pseudomonas aeruginosa	8.9	2.88	3.13
Escherichia coli	0.089	0	0
Salmonella enterica	0.089	0.04	0.04
Lactobacillus fermentum	0.0089	0	Not in database
Enterococcus faecalis	0.00089	0	0
Staphylococcus aureus	0.000089	0	0
Listeria monocytogenes	89.1	56.63	67.33
Bacillus subtilis	0.89	0.83	0.5
Saccharomyces cerevisiae	0.89	Not in database	Not in database
Cryptococcus neoformans	0.00089	Not in database	0
Unclassified	NA	34.78	28.14

Kraken2 with our custom databases can accurately identify the top ten pathogenic species found in clinical urine and skin swab samples at the HfSA between 2018 and 2019, with minimal cross-classification

Having shown that Kraken2 could identify species in a complex microbial community with our two custom databases, we next wanted to test its performance in samples more representative of those that would be sequenced when using our protocol for rapid diagnostics in a veterinary clinical setting (that is, mostly single pathogen infections). In the main text, we identified the top ten species that were found in canine urine and skin swab samples over a two year period at the Hospital for Small Animals in Edinburgh, and showed that our extraction protocol could extract sequenceable DNA from them all, including Gram positives, Gram negatives, aerobes, anaerobes, fast growers and slow growers. These ten species represent around 90% of all cases seen in clinical practice over that two year period; the remaining 10% consisted of several dozen species which were seen on only one or two occasions, hence validating our protocol on them all was unfeasible at this point. Here, we used the same ten species, plus *Salmonella enterica* to determine whether Kraken2 with our custom databases could identify some of the most commonly seen pathogens in canine urine and skin infections.

We downloaded existing nanopore sequencing data for eight of the ten species, where possible picking samples for which metadata was available in sufficient detail to determine sequencing characteristics such as flow cell version, library preparation kit (although this was deemed to be less important with regards to the eventual sequence data quality), sequencer and basecaller. For most of the samples, flow cell version and sequencer details were available, although basecaller and basecaller version were not. However, we know that the R9.4.1 flow cells plus basecalling with Guppy (v6.4.3) in super accuracy mode tend to give a median read quality score of 12.5-14 for our clinical samples (data not shown here). Therefore, we used NanoStat to conduct read QC and determine the median quality scores, as a proxy for knowing which basecaller was used. For two species, nanopore reads were not available from the NCBI's SRA. For these two (*P. canis and K. rosea*), a read simulation software, Badread, was used to simulate reads with an error profile matched to that of nanopore sequencing circa 2020. Species identification was then performed for each read set using Kraken2 with both custom databases, with a confidence threshold of 0.05. The % of reads assigned to the correct species was recorded, and any additional species to which >1% reads had been assigned were noted.

Table 6 shows the results of this testing. For both databases, at a confidence threshold of 0.05, the expected species was the most commonly identified for all species tested. The proportion of reads assigned to the correct species ranged from 2.87 (*E. coli*) to 92.44% (*Staphylococcus pseudintermedius*) for the bacteria_plus database, and from 0.86% (*Kocuria rosea*) to 95.04% (*Staphylococcus pseudintermedius*) for the pathogens_plus database. For both databases, nine of the 11 species tested were identified in >45% abundance. *E. coli* performed predictably poorly with the bacteria_plus database (2.87%), but relatively well with the pathogens_plus database (38.73%), whilst the opposite was true for *K. rosea* (33.05% with bacteria_plus, 0.86% with pathogens_plus). The reasons for *E. coli*'s poor performance were discussed above; in this case, reads were often assigned to other *Escherichia* or *Shigella* species, with 26% of reads classified as *Escherichia/Shigella* with bacteria_plus, and 74.31% classified as *Escherichia/Shigella* with pathogens_plus. Reassuringly, *E. coli* was always identified as the most abundant *Escherichia/Shigella* species, albeit at low abundance. The reasons for *K. rosea*'s poor performance with both databases, but particularly pathogens_plus, were less clear. However, this is a poorly studied (and therefore poorly sequenced) species, and it is possible that the reference genome included in the databases is not a good representative.

In addition, our results show a number of species which may be identified as false positives during sequencing. With pathogens_plus, incorrect species were only identified at an abundance of >1% for *E. coli*, suggesting a very low likelihood of this database producing false positives when sequencing the other species tested (or, presumably, other non-*Escherichia* species that might occur in our clinical samples). With bacteria_plus, three species (*E. coli*, *Streptococcus canis* and *Klebsiella pneumoniae*) resulted in the classification of a variety of incorrect species at an abundance >1%. One species seen in the *E. coli* sample (*Nocardioides alcanivorans*) frequently occurs in our negative control samples and we suspect it is a signal of background contamination (likely from tubes, as we see it more frequently with some brands than with others). Interestingly, with pathogens_plus, this same signal is usually seen as *Escherichia marmotae*. Encouragingly, even when other species were classified alongside the correct species, the correct species was always seen at by far the greatest abundance. Our results here can therefore be used in the interpretation of future classifications, in order to distinguish between true co-infections with two very similar species, and monopathogenic infections with a small degree of taxonomic misclassification in the background.

Overall, our results from this work support the use of both databases during classification, followed by manual comparison and interpretation of the results from both. If results are still ambiguous after manual interpretation, another round of Kraken2 testing could be performed after genome assembly, using the assembled contigs as reads.

Future improvements to read accuracy and refinements to our species classification strategies

As seen in **Table 2**, the median read quality scores in our downloaded and simulated samples ranged from 9.8 to 14.9; therefore, these samples were largely representative of the kinds of error profiles we would see when sequencing clinical samples with R9.4.1 flows cells and basecalling with Guppy (v6.4.3) in super accuracy mode. In fact, most of the downloaded and simulated reads (including both ZymoBIOMICS data sets) were of lower accuracy than the data we can currently produce with R9.4.1 flow cells, due to major improvements in more recent versions of Guppy. We are therefore confident that data produced using our rapid sequencing pipeline will produce species classifications at least as accurate, if not more accurate, than the data sets tested here. Using the information produced here, including the use of the confidence 0.05 threshold, we can produce results with minimal false positives and minimal false negatives, albeit with a tendency to underestimate the true abundance of the true positives (for this reason our test cannot currently be considered "quantitative"). In the future, newer flow cells (R10.4.1) and kits will likely be used, alongside future updates to basecalling software; the improved accuracy of these will presumably also improve the accuracy of our taxonomic classification with Kraken2.

With regards to future developments to our species classification strategies, we ultimately aim to be able to automate the analysis and interpretation; at present, our results require an expert eye to be sure the interpretation is free of false positives and negatives. This is not ideal for a clinical environment, where a diagnostics pipeline may not always be run by someone who is an expert in the bioinformatics of taxonomic classification. Therefore, one of our major future goals will be to sequence a larger number of known clinical samples, in order to better quantify what a "true positive" looks like with our databases, and whether the threshold of 1% of microbial reads is appropriate for all sample types. In addition, based on the results of a recent benchmarking study (5), we will test whether the addition of Bracken after Kraken2 classification improves classification accuracy enough to justify the additional time it would add to diagnosis.

Table 6: Kraken2 results for the various cultured species isolates with our custom databases, at confidence threshold 0.05, including any other species seen at >1% abundance

	Confidence 0.05					
	Bacteria_plus %			Pathogens_plus %		
**Pure sequenced isolate**	**Target species**	**Other species >1%**	**Unclassified reads**	**Target species**	**Other species >1%**	**Unclassified reads**
Escherichia coli	2.87	*Escherichia fergusonii* (2.15%), *Klebsiella michiganensis* (1.06%), *Nocardioides alcanivorans* (1.19%)	9.93	38.73	*Escherichia marmotae* (5.00%), *Escherichia ruysiae* (1.79%), *Escherichia fergusonii* (1.73%)	8.37
Pseudomonas aeruginosa	79.83	NA	9.59	83.16	NA	14.87
Salmonella enterica	58.77	NA	24.93	68.07	NA	25.26
Staphylococcus pseudintermedius	92.44	NA	2.22	94.05	NA	2.77
Streptococcus canis	80.32	*Streptococcus pseudoporcinus*	7.46	91.33	NA	6.03
Enterococcus faecalis	80.58	NA	12.44	79.68	NA	16.46
Proteus mirabilis	75.81	NA	13.8	79.27	NA	17.55
Pasteurella canis	59	NA	35.73	52.97	NA	43.42
Klebsiella pneumoniae	64.11	*Klebsiella quasivariicola* (1.38%), *Pseudomonas yangonensis* (1.42%), *Limoniibacter endophyticus* (2.80%)	4.03	78.18	NA	15.85
Kocuria rosea	33.05	NA	30.78	0.86	NA	50.6
Clostridium perfringens	45.18	NA	50.79	47.43	NA	50.03
Escherichia/Shigella	26	*Klebsiella michiganensis* (1.06%) *Nocardioides alcanivorans* (1.19%)	9.93	74.31	NA	8.37

References

1. Wood DE, Lu J, Langmead B. Improved metagenomic analysis with Kraken 2. Genome Biology. 2019;20(1):257.
2. Wood DE, Salzberg SL. Kraken: ultrafast metagenomic sequence classification using exact alignments. Genome Biol. 2014;15(3):R46.
3. Wright RJ, Comeau AM, Langille MGI. From defaults to databases: parameter and database choice dramatically impact the performance of metagenomic taxonomic classification tools. Microbial Genomics. 2023;9(3).
4. McIntyre KM, Setzkorn C, Hepworth PJ, Morand S, Morse AP, Baylis M. A Quantitative Prioritisation of Human and Domestic Animal Pathogens in Europe. PloS one. 2014;9(8):e103529.
5. Govender KN, Eyre DW. Benchmarking taxonomic classifiers with Illumina and Nanopore sequence data for clinical metagenomic diagnostic applications. Microbial Genomics. 2022;8(10).
6. Lu J, Rincon N, Wood DE, Breitwieser FP, Pockrandt C, Langmead B, et al. Metagenome analysis using the Kraken software suite. Nature protocols. 2022;17(12):2815-39.
7. Lu J, Breitwieser FP, Thielen P, Salzberg SL. Bracken: estimating species abundance in metagenomics data. PeerJ Computer Science. 2017;3(e104).
8. Nicholls SM, Quick JC, Tang S, Loman NJ. Ultra-deep, long-read nanopore sequencing of mock microbial community standards. GigaScience. 2019;8(5):giz043.
9. Hall MB. Rasusa: Randomly subsample sequencing reads to a specified coverage. Journal of Open Source Software. 2022;7(69):3941.
10. Wick RR. Badread: simulation of error-prone long reads. Journal of Open Source Software 2019;4(36):1316.
11. De Coster W, D'Hert S, Schultz DT, Cruts M, Van Broeckhoven C. NanoPack: visualizing and processing long-read sequencing data. Bioinformatics (Oxford, England). 2018;34(15):2666-9.
12. Ye SH, Siddle KJ, Park DJ, Sabeti PC. Benchmarking Metagenomics Tools for Taxonomic Classification. Cell. 2019;178(4):779-94.
13. Geurtsen J, de Been M, Weerdenburg E, Zomer A, McNally A, Poolman J. Genomics and pathotypes of the many faces of Escherichia coli. FEMS Microbiology Reviews. 2022;46(6):fuac031.
14. Portik DM, Brown CT, Pierce-Ward NT. Evaluation of taxonomic classification and profiling methods for long-read shotgun metagenomic sequencing datasets. BMC Bioinformatics. 2022;23(1):541.
15. Charalampous T, Alcolea-Medina A, Snell LB, Alder C, Tan M, Williams TGS, et al. Routine respiratory metagenomics service for intensive care unit patients. medRxiv. 2023:2023.05.15.23289731.
16. Charalampous T, Alcolea-Medina A, Snell LB, Williams TGS, Batra R, Alder C, et al. Evaluating the potential for respiratory metagenomics to improve treatment of secondary infection and detection of nosocomial transmission on expanded COVID-19 intensive care units. Genome Medicine. 2021;13(1):182.
17. Cheng H, Sun Y, Yang Q, Deng M, Yu Z, Zhu G, et al. A rapid bacterial pathogen and antimicrobial resistance diagnosis workflow using Oxford nanopore adaptive sequencing method. Briefings in bioinformatics. 2022;23(6):bbac453.
18. Lin Y, Dai Y, Zhang S, Guo H, Yang L, Li J, et al. Application of nanopore adaptive sequencing in pathogen detection of a patient with Chlamydia psittaci infection. Front Cell Infect Microbiol. 2023;13:1064317.
19. Marchukov D, Li J, Juillerat P, Misselwitz B, Yilmaz B. Benchmarking microbial DNA enrichment protocols from human intestinal biopsies. Front Genet. 2023;14:1184473.
20. Martin S, Heavens D, Lan Y, Horsfield S, Clark MD, Leggett RM. Nanopore adaptive sampling: a tool for enrichment of low abundance species in metagenomic samples. Genome Biology. 2022;23(1):11.

21. Su J, Lui WW, Lee Y, Zheng Z, Siu GK-H, Ng TT-L, et al. Evaluation of Mycobacterium tuberculosis enrichment in metagenomic samples using ONT adaptive sequencing and amplicon sequencing for identification and variant calling. Scientific reports. 2023;13(1):5237.
22. Marquet M, Zöllkau J, Pastuschek J, Viehweger A, Schleußner E, Makarewicz O, et al. Evaluation of microbiome enrichment and host DNA depletion in human vaginal samples using Oxford Nanopore's adaptive sequencing. Scientific reports. 2022;12(1):4000.
23. Viehweger A, Marquet M, Hölzer M, Dietze N, Pletz MW, Brandt C. Adaptive nanopore sequencing on miniature flow cell detects extensive antimicrobial resistance. bioRxiv. 2022:2021.08.29.458107.
24. Wick RR. Filtlong: quality filtering tool for long reads 2017 [Available from: https://github.com/rrwick/Filtlong.

Supplementary Results SR2

Testing of adaptive sampling to reduce host DNA contamination

Background

Clinical samples, including both urine and skin swabs, may have high levels of host DNA alongside any pathogens. The pipeline developed here is primarily aimed at processing clinical samples and it is therefore likely that many of the DNA samples extracted will contain host DNA alongside the bacterial DNA. In some cases, such as purulent skin swabs or urine samples containing high numbers of white blood cells, the vast majority of the DNA extracted may be derived from the host. For example, the urine samples we sequenced in this manuscript were, on average, around 50% dog DNA, but the skin swab samples were usually at least 95% dog DNA, and often >99% dog DNA. As discussed in the main text, extremely high levels of host DNA in a sample, like those seen in our skin swab samples, can prevent accurate prediction of AMR sensitivity, because most of the DNA sequenced is dog, thus too little bacterial genome coverage is produced, and genes may be missed. Due to the relatively manageable levels of host DNA seen in urine samples, pre-sequencing methods to reduce host DNA, such as saponin-plus-DNase treatment or PMA-plus-UV-light treatment, were not explored here. As an alternative, a sequencing-based sampling method was trialled.

Adaptive sampling is a technique unique to nanopore-based sequencers, in which DNA reads are compared to a reference genome whilst the strand of DNA is still being sequenced. The technique can be used either to enhance or to deplete sequences which map to the reference genome, according to which option the user has selected. If “deplete” is selected, when a read being sequenced maps to the chosen reference genome, the ionic current across the relevant pore is briefly reversed, ejecting the DNA strand and therefore not sequencing any more of it. If “enhance” is selected the opposite happens, and all reads which do not map to the reference genome are ejected. Due to the computer power needed to do this in real-time, MinION sequencers are unlikely to be capable of adaptive sampling, unless connected to a GPU-powered computer. The GridION sequencer, however, is equipped with GPU processors, and adaptive sampling is therefore possible. Although the option of performing adaptive sampling during sequencing has only been available on the software which controls nanopore sequencers (MinKNOW) for a relatively short time, a number of studies have already investigated its use for both enrichment and depletion in metagenomic samples, usually with promising results (17-23).

The purpose of the additional work detailed in this supplementary document was to test the effectiveness of adaptive sampling on a variety of levels of dog DNA contamination, using a single MinION flow cell sequenced on our GridION.

Methods

Source code and full commands used for mentioned data analysis tools are available from Github: https://github.com/nataliering/Dogstails

Two DNA samples were used to test the efficiency of adaptive sampling for reducing levels of host DNA in clinical samples: the same *S. pseudintermedius* isolate (ED99) and healthy dog DNA used for the section "Determining the lower detection limits for the rapid barcoding (SQK-RBK004) kit" in the main text. The 75 ng μl^{-1} samples were mixed in various ratios (90:10, 75:25, 50:50, 25:75 and 10:90, **Table 1**). 7.5 µl of each mix was prepared for sequencing with SQK-RBK004, each with a different barcode. Nuclease-free water with barcode01 was used as a negative control, whilst 100% dog and 100% *S. pseudintermedius* were used a two positive controls, each also with different barcodes.

The pooled prepared library was then sequenced on a fresh R9.4.1 flow cell on a GridION, with real-time super accuracy basecalling (Guppy v6.4.6, GridION software release v22.12.5). The run was set up via MinKNOW so that half of the flow cell's channels (channels 1-256) were sequencing as normal, whilst the other half (channels 257-512) were running adaptive sampling to deplete DNA which mapped to a canine reference genome provided to the software (GCA_014441545.1 ROS_Cfam_1.0).

Table 1: Samples sequenced during adaptive sampling trial

**Sample**	Ratio dog DNA : bacterial DNA
A	0:0 (negative control)
B	100:0
C	90:10
D	75:25
E	50:50
F	25:75
G	10:90
H	0:100

The reads sequenced by channels 1-256 (without adaptive sampling) were separated from those sequenced by channels 257-512 (with adaptive sampling) informatically (see Github for commands). Read QC was carried out with NanoStat (v1.6.0, 11). The resulting datasets for each barcode were analysed using Kraken2 (v2.1.1, 1) with the bacteria_plus database described above. The percentage of reads from each sample assigned to *Staphylococcus pseudintermedius* and *Canis lupus familiaris*

were recorded, and the differences between the percentages of each with and without adaptive sampling were compared. Later, Filtlong (v0.2.0, 24) was used to filter out reads shorter than 1,000 bp, Kraken2 was re-run, and the results for the >1,000 bp filtered datasets were also compared with the original, unfiltered datasets.

Results and Discussion

Several factors can influence the efficiency of adaptive sampling. DNA strands need to be long enough that they can be basecalled and mapped to a reference genome before the entire strand has traversed through the pore, in order for it to be ejected. This means DNA strands of at least hundreds, if not thousands of bases long. Some DNA extracted using physical lysis (e.g. bead bashing) may not sequence very efficiently with adaptive sampling. Likewise, the bigger the reference genome, the longer it may take to fully map a read. For a relatively large mammalian reference genome (like the 2.5 Gb *Canis lupus familiaris* genome), the DNA strand being sequenced may have fully traversed through the pore before the basecalled read can be mapped to the whole genome, meaning it can no longer be ejected even if a positive hit was found. Finally, basecalling and mapping speed are highly important. The GridION is usually capable of real-time super accuracy basecalling with Guppy with a single flow cell being sequenced, but basecalling may lag if more DNA is being sequenced (e.g. if a single flow cell has very high pore occupancy, or if multiple flow cells are being sequenced simultaneously). As we are generally sequencing low concentration DNA samples on single flow cells with low pore occupancy, and our read N50 is usually >5,000 bp, our major concerns for adaptive sampling was neither basecalling speed nor strand length, but the size of the canine reference genome we were using to deplete host DNA.

We compared the differences in the percentages of dog and *S. pseudintermedius* DNA in our samples with and without adaptive sampling, and without any read length filtering, **Figure 1A, Table 2**. We can see without any read length filtering, there appears to be very limited difference in the percentage of reads in each sample assigned to dog or *S. pseudintermedius* with and without adaptive sampling, ranging from -0.42% to + 0.03% for dog (mean= -0.64%), and from -0.01% to +1.43% for *S. pseudintermedius* (mean= +0.54%). Although the adaptive sampling has on average reduced the percentage of reads assigned to dog and increased the percentage assigned to *S. pseudintermedius*, the different is very small, and unlikely to improve ability to identify AMR genes.

However, our read QC indicated significantly different read length distributions in the read sets with and without adaptive sampling; **Figure 3** shows this effect for sample B (100% dog DNA). The peak of the read length histogram with adaptive sampling was smaller than 1,000 bp, whereas the peak without adaptive sampling was closer to 10,000 bp. We suspected this indicated that the adaptive

A)

B)

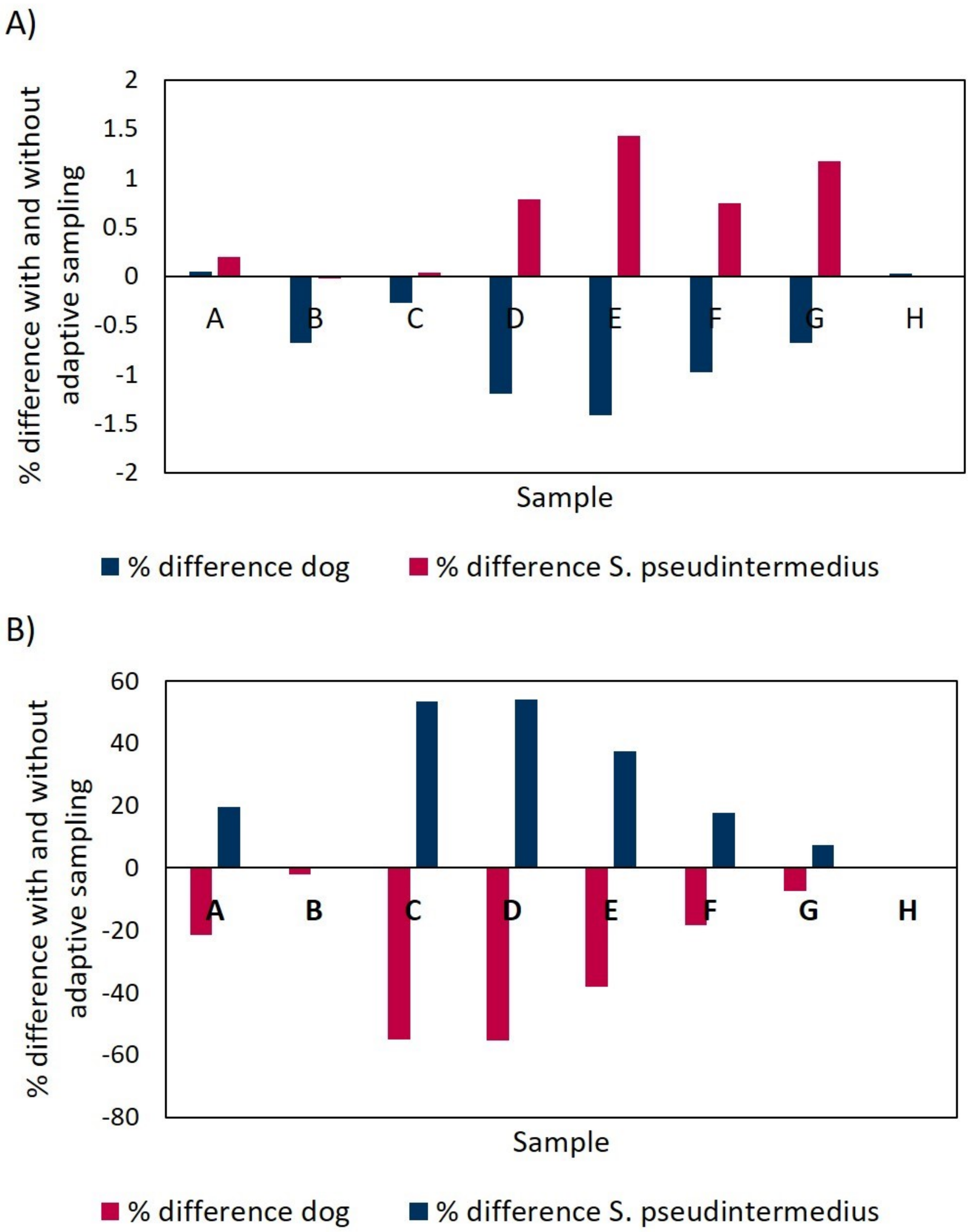

Figure 5: the difference in % of each sample assigned to "*Canis lupus familiaris*" and *"Staphylococcus pseudintermedius*" by Kraken2 with our bacteria_plus custom database when sequencing with and without adaptive sampling. Samples A-H ranged from 100% dog to 0% dog, as shown in **Table 1.** A) results without read length filtering, B) results with reads <1,000 bp filtered out using Filtlong

sampling was indeed having an effect: the shorter reads were the fragments of DNA which traversed the pore and were basecalled before the rest of the longer strand was ejected. If correct, this would mean that all of the shorter read were dog, and the host DNA could therefore potentially be excluded by using read length as a proxy.

We tested this theory by using Filtlong to filter out all reads shorter than 1,000 bp in samples A-H, and re-ran our analysis with Kraken2. **Figure 1B** and **Table 3** show the differences in the percentages of dog and *S. pseudintermedius* DNA in our samples with and without adaptive sampling after filtering out reads shorter than 1,000 bp. After removing the shorter reads, the apparent effectiveness of the adaptive sampling has increased considerably: the percent difference ranged from -0.08% to -55.41% for dog (mean= -24.78%) and from -0.27% to +54.04% (mean= +23.66%). The most promising results were for the samples with the highest starting proportions of dog DNA, samples C and D with 90 and 75% starting dog DNA respectively; with adaptive sampling, sample C had 55.01% less dog DNA and 53.52% more *S. pseudintermedius* DNA, whilst sample D had 55.41% less dog DNA and 54.05% more *S. pseudintermedius* DNA. Interestingly, filtering out reads shorter than 1,000 bp also removed the vast majority of background contamination seen in our negative control (sample A). We also compared the total amount of *S. pseudintermedius* DNA sequenced with and without adaptive sampling, to determine whether adaptive sampling actually increased the absolute amount of bacteria sequenced and not just the amount relative to dog. **Figure 3** indicates that the absolute amount of bacterial DNA being sequenced was indeed increased by adaptive sampling, with differences ranging from -0.12 Mb to 125.26 Mb (mean= +44.72 Mb). The difference for sample C, which started with 90% dog DNA, was fairly small at 14 Mb, although the difference for sample D (75% dog) was more significant, at 63.49%.

Filtering out reads <1,000 bp is not supposed to be required in order to see the effects of adaptive sampling. We believe this was necessary for our samples due to the larger size of the canine reference genome, which allowed the sequencing of several hundreds of base pairs of dog DNA prior to the strand being mapped and subsequently ejected. Our results suggest that adding a step to filter out shorter reads could significantly reduce the levels of dog DNA contamination in our clinical samples, including those with the highest levels (>90%), as well as all but eliminating “kitome” background contamination DNA. We believe the results of this simple trial of adaptive sampling suggest that, combined with pre- or post-extraction host depletion steps, adaptive sampling could significantly reduce the levels of host DNA contamination in clinical samples, thereby improving the accuracy of our AMR predictions. Where a GridION (or GPU computer plus MinION) is available, we would therefore recommend the use of adaptive sampling in our protocol.

Table 2: Run results with and without adaptive sampling without read length filtering

		Without adaptive sampling				With adaptive sampling			
**Sample**	**Ratio (dog:bacteria)**	**Total reads**	**Run yield (Mb)**	**% dog**	**% *S. pseudintermedius***	**Total reads**	**Run yield (Mb)**	**% dog**	**% *S. pseudintermedius***
A	0:0	1600	0.7	1.69	0.69	2012	0.74	1.74	0.89
B	100:0	78774	428	98.18	0.03	116426	67.54	97.5	0.01
C	90:10	53615	341.8	84.58	13.03	78,260	122	84.31	13.07
D	75:25	50419	371.6	68.48	29.63	73,850	233.24	67.28	30.41
E	50:50	26784	213.6	43.75	53.66	39644	210.84	42.33	55.09
F	25:75	15361	142.4	20.62	76.09	22451	177.8	19.64	76.83
G	10:90	23833	236.7	8.02	87.67	35816	349.7	7.34	88.84
H	0:100	7070	64.2	0.08	95.19	9899	94.4	0.11	95.18

Table 3: Run results with and without adaptive sampling without reads <1,000 bp filtered out

		Without adaptive sampling				With adaptive sampling			
**Sample**	**Ratio (dog:bacteria)**	**Total reads**	**Run yield (Mb)**	**% dog**	**% *S. pseudintermedius***	**Total reads**	**Run yield (Mb)**	**% dog**	**% *S. pseudintermedius***
A	0:0	60	0.3	35.00	15.00	52	0.2	13.46	34.62
B	100:0	60695	418.2	99.71	0.03	6768	12.9	97.61	0.01
C	90:10	44616	336.7	85.81	13.68	13501	89.4	30.80	67.20
D	75:25	43441	367.5	68.57	30.51	23854	207.7	13.16	84.55
E	50:50	23278	211.5	43.42	55.15	21163	201.2	5.22	92.52
F	25:75	13431	141.3	20.15	77.99	16235	174.5	1.75	95.59
G	10:90	20980	235.1	8.07	89.47	29704	346.4	0.59	96.89
H	0:100	5973	63.6	0.10	97.42	8523	93.6	0.02	97.15

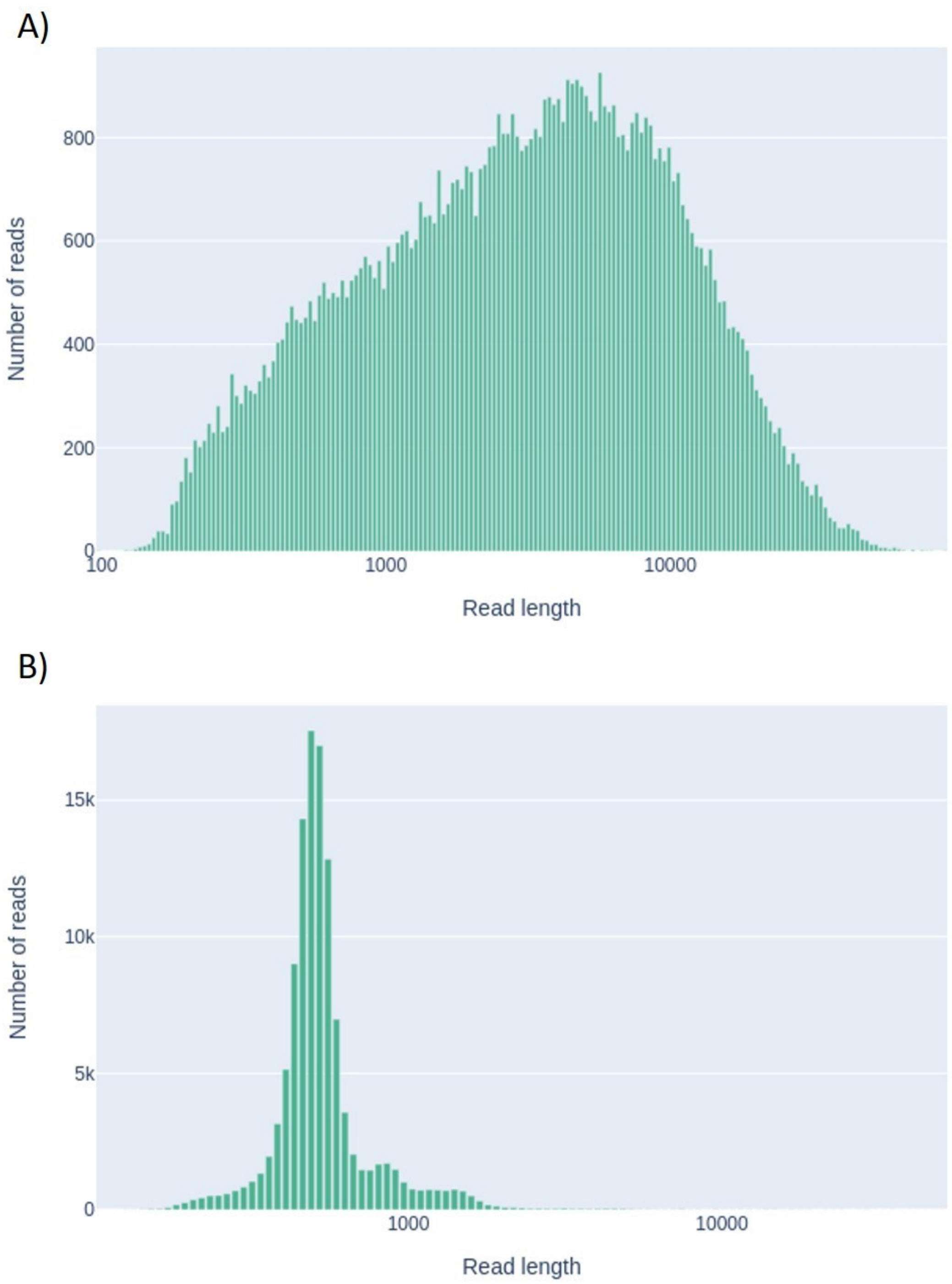

Figure 6: Read length distribution for sample B (100% dog DNA). A) without adaptive sampling. B) with adaptive sampling. Non-weighted histograms with log transformation produced with NanoStat.

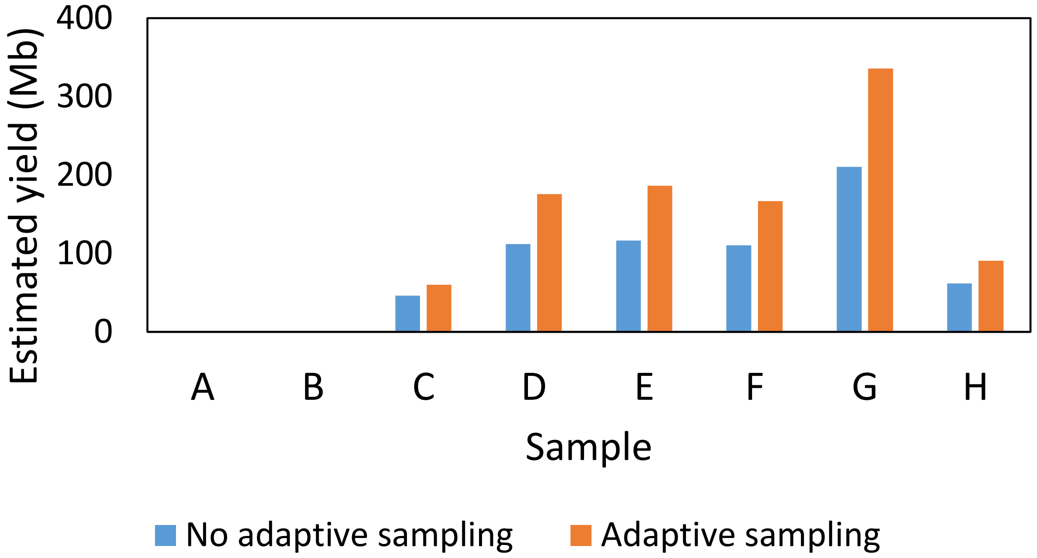

Figure 7: The yield of *S. pseudintermedius* DNA in samples A-H with and without adaptive sampling. Yield in base pairs was estimated by multiplying the percentage of reads called as *S. pseudintermedius* by Kraken2 by the read length N50.

References

1. Cheng H, Sun Y, Yang Q, Deng M, Yu Z, Zhu G, et al. A rapid bacterial pathogen and antimicrobial resistance diagnosis workflow using Oxford nanopore adaptive sequencing method. Briefings in bioinformatics. 2022;23(6):bbac453.
2. Lin Y, Dai Y, Zhang S, Guo H, Yang L, Li J, et al. Application of nanopore adaptive sequencing in pathogen detection of a patient with Chlamydia psittaci infection. Front Cell Infect Microbiol. 2023;13:1064317.
3. Marchukov D, Li J, Juillerat P, Misselwitz B, Yilmaz B. Benchmarking microbial DNA enrichment protocols from human intestinal biopsies. Front Genet. 2023;14:1184473.
4. Martin S, Heavens D, Lan Y, Horsfield S, Clark MD, Leggett RM. Nanopore adaptive sampling: a tool for enrichment of low abundance species in metagenomic samples. Genome Biology. 2022;23(1):11.
5. Su J, Lui WW, Lee Y, Zheng Z, Siu GK-H, Ng TT-L, et al. Evaluation of Mycobacterium tuberculosis enrichment in metagenomic samples using ONT adaptive sequencing and amplicon sequencing for identification and variant calling. Scientific reports. 2023;13(1):5237.
6. Marquet M, Zöllkau J, Pastuschek J, Viehweger A, Schleußner E, Makarewicz O, et al. Evaluation of microbiome enrichment and host DNA depletion in human vaginal samples using Oxford Nanopore's adaptive sequencing. Scientific reports. 2022;12(1):4000.
7. Viehweger A, Marquet M, Hölzer M, Dietze N, Pletz MW, Brandt C. Adaptive nanopore sequencing on miniature flow cell detects extensive antimicrobial resistance. bioRxiv. 2022:2021.08.29.458107.
8. De Coster W, D'Hert S, Schultz DT, Cruts M, Van Broeckhoven C. NanoPack: visualizing and processing long-read sequencing data. Bioinformatics (Oxford, England). 2018;34(15):2666-9.
9. Wood DE, Lu J, Langmead B. Improved metagenomic analysis with Kraken 2. Genome Biology. 2019;20(1):257.
10. Wick RR. Filtlong: quality filtering tool for long reads 2017 [Available from: https://github.com/rrwick/Filtlong.
